# Reevaluating the senolytic activity of a GLS1 inhibitor and an anti-PD-1 antibody: toward greater reproducibility and methodological rigor

**DOI:** 10.1038/s44319-026-00740-5

**Published:** 2026-04-03

**Authors:** Shimpei Kawamoto, Haruki Horiguchi, Daisuke Torigoe, Masahiro Wakita, Koyu Ito, Sho Sugawara, Xiangyu Zhou, Takumi Mikawa, Jeong Hoon Park, Birte Kristin Jung, Yumiko Okumura, Hideka Miyagawa, Mikako Maruya, Nozomi Hori, Ken Uemura, Masataka Sugimoto, Michiyuki Matsuda, Naoki Mochizuki, Hiroshi Kondoh, Akiko Takahashi, Yuichi Oike, Eiji Hara

**Affiliations:** 1 Research Institute for Microbial Diseases, The University of Osaka, Suita, Japan; 2 Institute of Development, Aging and Cancer, Tohoku University, Sendai, Japan; 3 Organization for Advanced Studies, Tohoku University, Sendai, Japan; 4 Graduate School of Medical Sciences, Kumamoto University, Kumamoto, Japan; 5 Institute of Resource Development and Analysis, Kumamoto University, Kumamoto, Japan; 6 Cancer Institute, Japanese Foundation for Cancer Research, Tokyo, Japan; 7 Kyoto University Hospital, Kyoto, Japan; 8 RIKEN Center for Integrative Medical Sciences, Yokohama, Japan; 9 National Center for Geriatrics and Gerontology, Obu, Japan; 10 Graduate School of Medicine, Kyoto University, Kyoto, Japan; 11 Research Institute, National Cerebral and Cardiovascular Center, Suita, Japan; 12 Immunology Frontier Research Center, The University of Osaka, Suita, Japan

**Keywords:** Cellular Senescence, Senolysis, Senolytic Drug, Aging, Reproducibility, Autophagy & Cell Death, Molecular Biology of Disease, Pharmacology & Drug Discovery

## Abstract

The discovery of the senescence-associated secretory phenotype (SASP) has reshaped our understanding of cellular senescence, shifting its role from a solely tumor-suppressive mechanism to a potential driver of chronic inflammation and age-related diseases. Accordingly, senolytic drugs, which selectively eliminate senescent cells, have garnered considerable interest due to promising preclinical studies. However, concerns remain regarding the reproducibility and generalizability of these findings. In this cross-laboratory study, we rigorously tested the senolytic efficacy of a GLS1 inhibitor and an anti-PD-1 antibody—agents previously reported to reduce the burden of p16^INK4a^-positive senescent cells and improve health outcomes in aged mice. Contrary to earlier reports, our study demonstrates that neither GLS1 inhibition nor PD-1 blockade significantly reduced p16^INK4a^-positive cell burden or improved aging-related health parameters. Although we do not seek to discredit prior work, our results underscore the need for rigorous design, standardized protocols, and independent validation to ensure reliable senolytics before clinical translation.

## Synopsis

A cross-laboratory, blinded replication study of two previously reported senolytic interventions finds that neither the GLS1 inhibitor BPTES nor anti-PD-1 antibody treatment significantly reduces endogenous p16^INK4a^-positive cell burden or improves functional aging parameters in aged mice. These findings underscore the importance of rigorous, standardized, and independently validated experimental designs for evaluating senolytic therapies.

- In multiple human fibroblast models, BPTES kills senescent cells only at doses that also impair proliferation and survival of non-senescent cells.
- In both conventional and blinded cross-laboratory mouse studies, BPTES does not reduce endogenous p16^INK4a^ expression in liver, lung, or kidney, nor does it improve grip strength or external aging phenotypes.
- Anti-PD-1 antibody treatment or genetic PD-1 deletion fails to decrease p16^INK4a^ levels in aged mice, challenging prior claims of senolytic effects.

## Abstract (web summary)

A cross-laboratory, blinded replication study of two previously reported senolytic interventions finds that neither the GLS1 inhibitor BPTES nor anti-PD-1 antibody treatment significantly reduces endogenous p16^INK4a^-positive cell burden or improves functional aging parameters in aged mice. These findings underscore the importance of rigorous, standardized, and independently validated experimental designs for evaluating senolytic therapies.

## Graphical abstract

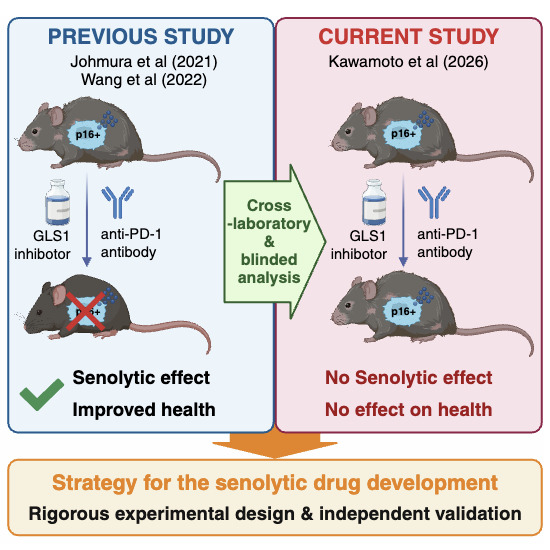

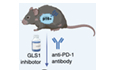

## Introduction

Cellular senescence is a state of stable cell-cycle arrest that can be triggered by various potentially oncogenic stimuli, including telomere erosion, radiation, oxidative stress, and oncogene activation (Campisi and d’Adda di Fagagna, 2007; Gorgoulis et al, 2019; Serrano and Blasco, 2001). Furthermore, many key genes involved in the induction of cellular senescence are classified as tumor suppressor genes, suggesting that cellular senescence plays a crucial role in tumor suppression (Collado and Serrano, 2010; He and Sharpless, 2017). Unlike apoptosis, however, senescent cells do not undergo immediate cell death; consequently, they gradually accumulate in the body with age (Krishnamurthy et al, 2004; Yamakoshi et al, 2009). Importantly, senescent cells are not simply arrested in the cell-cycle but also exhibit the senescence-associated secretory phenotype (SASP), characterized by the secretion of inflammatory cytokines, chemokines, growth factors, and extracellular matrix-degrading enzymes (Acosta et al, 2008; Coppe et al, 2008; Kuilman et al, 2008). Thus, excessive senescent cell accumulation can drive chronic inflammation in surrounding tissues through SASP, potentially contributing to functional decline and promoting the onset of various age-related diseases, including cancer (Chan and Narita, 2019; Lee and Schmitt, 2019; Watanabe et al, 2017). Indeed, van Deursen and colleagues developed a mouse model harboring a transgene in which an apoptosis-inducing gene is driven by the 2.7 kb promoter of *p16*^*INK4a*^, a key senescence-inducing gene (Baker et al, 2011). Using this model, they genetically demonstrated that the ablation of *p16*^*INK4a*^*-*expressing cells reduced the incidence of aging-associated cancers and extended health span (Baker et al, 2016). Since this report, the concept that eliminating senescent cells can extend health span has garnered significant attention (Naylor et al, 2013). Consequently, the development of senolytic drugs, which selectively eliminate senescent cells, has been actively pursued worldwide (Chaib et al, 2022; Wang et al, 2022; van Deursen, 2019). However, senescent cells exhibit considerable heterogeneity (Tao et al, 2024; Wechter et al, 2023), and some contribute beneficially to specific physiological processes, such as promoting wound healing, maintaining blood-tissue barriers, and activating immune responses, depending on the biological context (Grosse et al, 2020; Kang et al, 2011; Krizhanovsky et al, 2008; Reyes et al, 2022; Zhao et al, 2024). Therefore, the indiscriminate elimination of senescent cells may result in severe adverse effects, underscoring the need for a more selective and cautious approach in the development of senolytic drugs (Amor et al, 2020; Suda et al, 2021; Yoshida et al, 2020).

To date, more than 20 senolytic drug candidates have been reported, with some shown to improve health in aged mice (Chaib et al, 2022; Wang et al, 2022; Power et al, 2023). For example, BPTES, a GLS1 inhibitor, almost completely eliminated senescent cells in culture at a 10 μM concentration (Johmura et al, 2021). Furthermore, BPTES administration in aged mice reportedly reduced the burden of *p16*^*INK4a*^-expressing cells across multiple organs, resulting in significant improvements in health parameters (Johmura et al, 2021). However, our analysis comparing the activities and specificities of several senolytic drugs suggested that BPTES exhibits relatively low specificity and limited senolytic activity (Wakita et al, 2026). In biological experiments, subtle differences in experimental conditions and technique proficiency may influence the quality of the data. Therefore, we deemed it essential to evaluate these data in independent laboratories.

## Results and discussion

**Figure 1:**
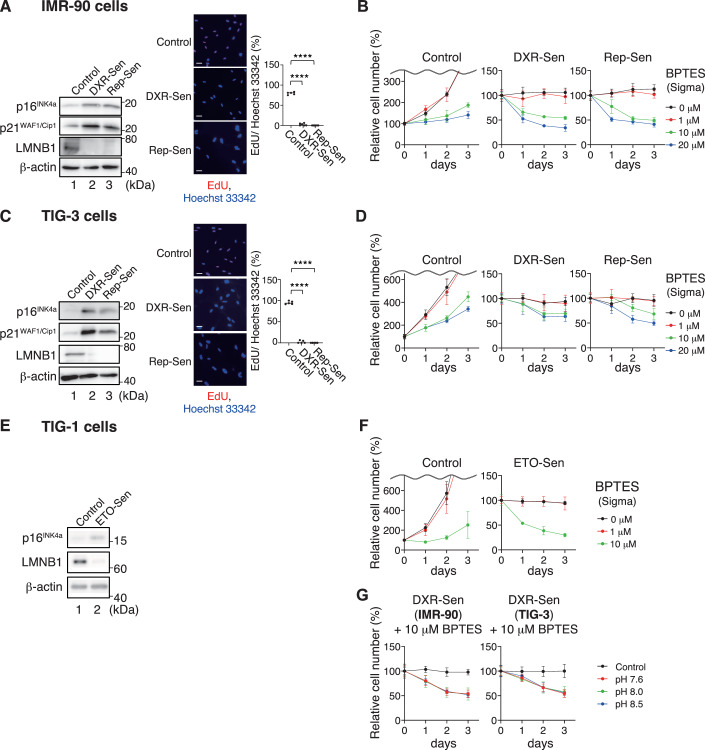
BPTES reduces proliferation and survival of cultured cells. (**A**–**G**) Early-passage (Control) IMR-90, TIG-3, and TIG-1 cells were rendered senescent by serial passaging (Rep-Sen), treatment with 250 ng/ml doxorubicin for 10 days (DXR-Sen), or treatment with 100 μM etoposide for 2 days followed by 10 days of culture in normal medium (ETO-Sen). These cells were subjected to western blot analysis using the indicated antibodies (**A**, **C**,** E**) and EdU incorporation assay (**A**,** C**) to confirm their senescent state. β-actin was used as a loading control (**A**,** C**,** E**). Quantification of EdU-positive cells is shown in the right panels (**A**,** C**). Control and senescent cells were treated with BPTES at the indicated concentrations for 3 days, and cell numbers were monitored throughout the experimental period (**B**,** D**,** F**). DXR-Sen IMR-90 and TIG-3 cells were treated with 10 μM BPTES under different pH conditions, and the number of surviving cells was counted. Control indicates senescent cells without BPTES treatment (**G**). Data are presented as mean ± s.d. (**A**,** C**: *n* = 5–6; **B**, **D**, **G**: *n* = 4; **F**: *n* = 3). All of the experiments were repeated at least twice, independently, with similar results. Statistical significance was determined by one-way ANOVA with Dunnett’s test (**A**, **C**). Control vs. DXR-Sen, ****p < 0.0001; Control vs. Rep-Sen, ****p < 0.0001 (**A**, **C**). Scale bars, 10 μm (**C**, **E**). Source data are available online for this figure.

**Figure EV1:**
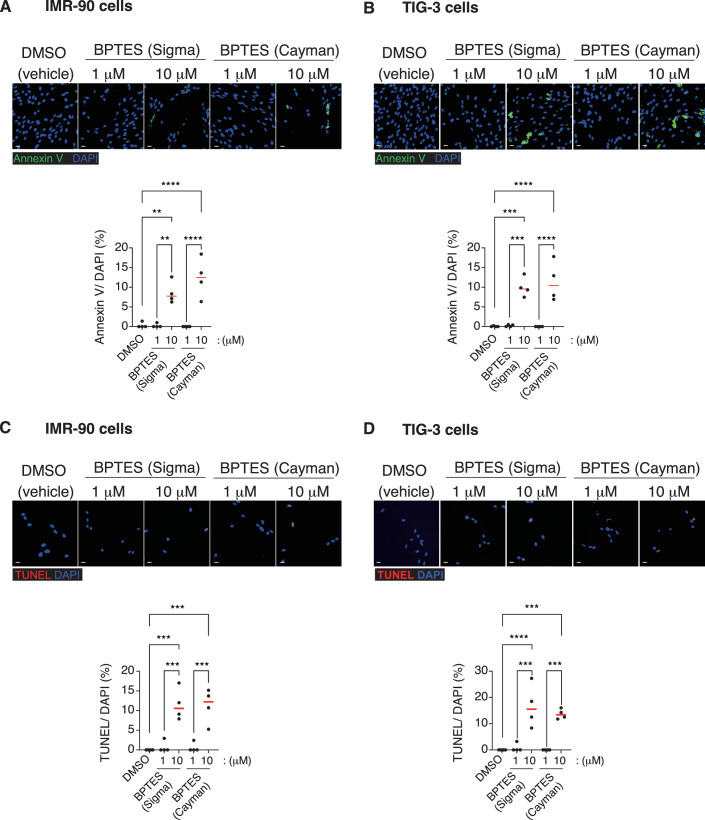
High dose of BPTES elicit apoptosis in control HDFs. (**A**–**D**) Non-senescent early-passage HDFs, IMR-90 (**A**,** C**) and TIG-3 (**B**,** D**), were treated with BPTES from Sigma-Aldrich or Cayman at the indicated concentrations for 2 days. Apoptotic cells positive for fluorochrome-labeled annexin V or TUNEL were visualized by fluorescence microscopy, and the proportions of annexin V- or TUNEL-positive cells were quantified. Data are presented as mean ± s.d. (**A**–**D**, *n* = 4). All of the experiments were repeated at least twice, independently, with similar results. Statistical significance was determined by one-way ANOVA followed by Sidak’s test. DMSO vs. BPTES (Sigma) 10 mM: **p = 0.0018 (IMR-90), ***p = 0.0002 (TIG-3); DMSO vs. BPTES (Cayman) 10 μM: ****p < 0.0001 (IMR-90, TIG-3); BPTES (Sigma) 1 vs. 10 μM: **p = 0.0016 (IMR-90), ***p = 0.0002 (TIG-3); BPTES (Cayman) 1 vs. 10 μM: ****p < 0.0001 (IMR-90, TIG-3) (**A**,** B**). DMSO vs. BPTES (Sigma) 10 μM: ***p = 0.0001 (IMR-90), ****p < 0.0001 (TIG-3); DMSO vs. BPTES (Cayman) 10 μM: ***p = 0.0002 (IMR-90), ***p = 0.0006 (TIG-3); BPTES (Sigma) 1 vs. 10 μM: ***p = 0.0003 (IMR-90), ***p = 0.0001 (TIG-3); BPTES (Cayman) 1 vs. 10 μM: ***p = 0.0003 (IMR-90), ***p = 0.0006 (TIG-3) (**C**,** D**). Scale bars, 10 μm. Source data are available online for this figure.

**Figure EV2:**
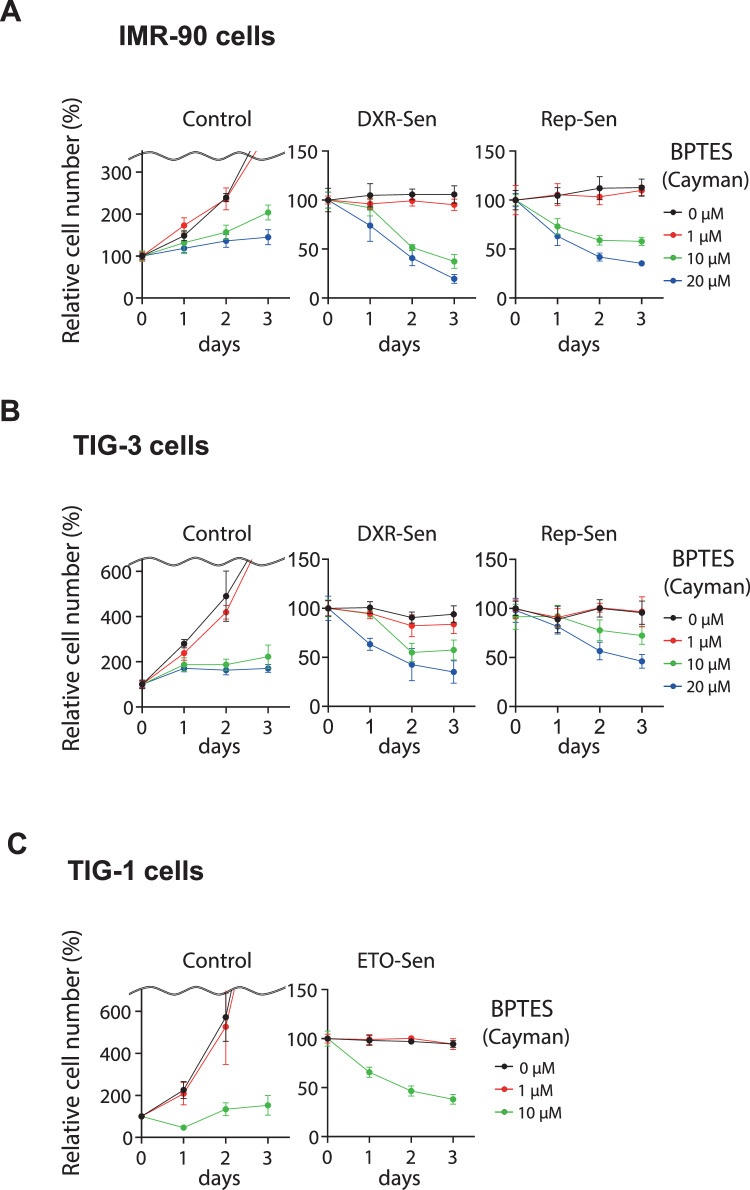
Effect of BPTES from different suppliers on proliferation and survival of senescent HDFs. (**A**–**C**) Control and senescent HDFs were treated with BPTES from Cayman at the indicated concentrations for 3 days. Cell numbers were monitored throughout the experimental period, and relative cell numbers were quantified. Data are presented as mean ± s.d. (**A**,** B**: *n* = 4; **C**: *n* = 3). All of the experiments were repeated at least twice, independently, with similar results. Source data are available online for this figure.

To validate the senolytic effects of BPTES, which appears to be a highly potent senolytic agent (Johmura et al, 2021), we conducted independent replication studies in the Hara (Osaka), Takahashi (Tokyo), and Kondoh (Kyoto) laboratories. Experiments conducted in the Hara and Takahashi laboratories using multiple cell types—including IMR-90 cells, a normal human diploid fibroblast (HDF) line also used by Johmura et al (2021)—revealed that treatment with BPTES (10 μM) resulted in cell death in approximately 50% of IMR-90 senescent cells and 30% of TIG-3 senescent cells, regardless of the method used to induce senescence (Fig. 1A–D). However, at this concentration, BPTES markedly inhibited the proliferation of non-senescent (control) cells and induced a certain degree of cell death (Figs. 1B,D and EV1). In Kondoh’s laboratory, similar results were observed in senescent TIG-1 cells—another type of HDF—induced by the DNA-damaging agent etoposide (Fig. 1E,F). Notably, increasing the concentration of BPTES to 20 μM led to a slight increase in senescent cell death. However, at this concentration, the proliferation of control cells was also more severely inhibited than at 10 μM (Fig. 1B,D). Conversely, although 1 μM BPTES had no growth-inhibitory effect on control cells, it also failed to induce cell death in senescent cells (Fig. 1B,D). Furthermore, given that a GLS1 inhibitor reportedly induced senescent cell death by impairing the neutralization of intracellular acidosis, we next examined whether an incubation in alkaline medium (pH 8.5), as described by Johmura et al (2021), could attenuate BPTES-induced senescent cell death. Contrary to their report (see Fig. 1G,H in Johmura et al, 2021), the cell death-inducing effect of 10 μM BPTES was completely unaffected by the incubation in alkaline medium (Fig. 1G). This result was consistently reproduced in independent experiments conducted in both Hara’s and Takahashi’s laboratories. Since differences between BPTES lots might have influenced the results, we tested six different lots from two independent suppliers. Nevertheless, our findings remained largely consistent across all batches (Figs. EV1 and EV2). Collectively, these results suggest that the GLS1 inhibitor BPTES does not exhibit truly selective toxicity toward senescent cells, prompting us to reevaluate the previous report claiming that BPTES administration reduces the p16^INK4a^-expressing cell population and ameliorates aging-related phenotypes in aged mice (Johmura et al, 2021).

**Figure 2:**
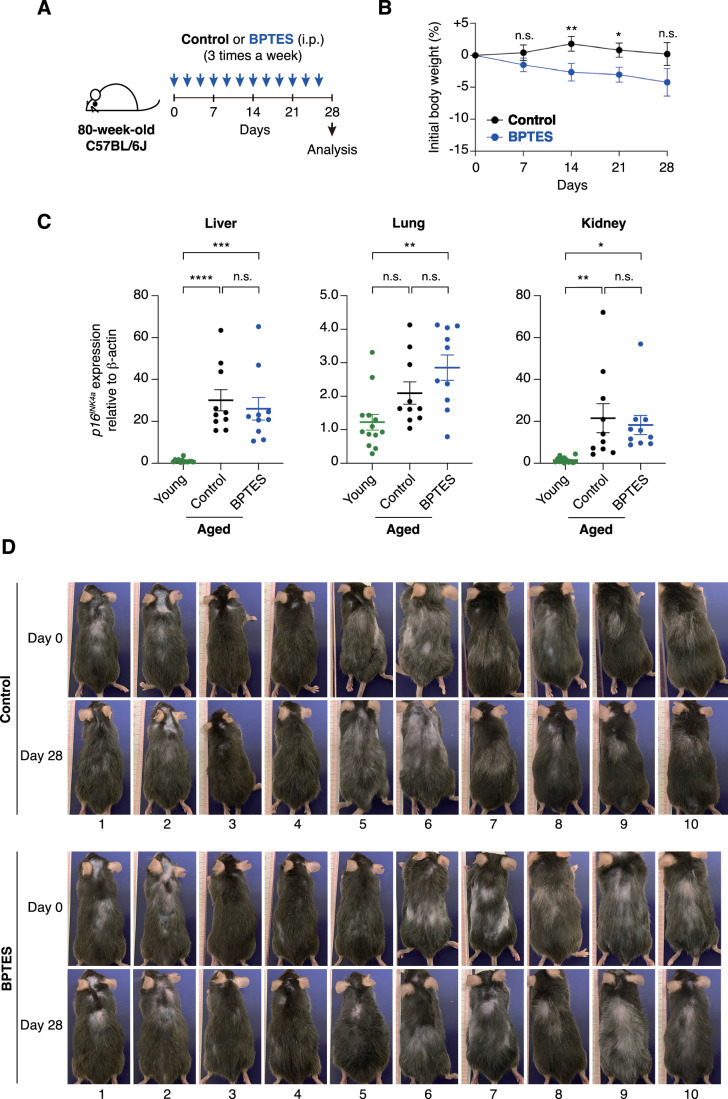
Administration of BPTES fails to reduce p16INK4a expression in aged mice. (**A**) Timeline of the experimental procedure for BPTES administration in 80-week-old C57BL/6 J mice. (**B**) Body weight changes over time, shown relative to the starting weight, in mice treated with BPTES or vehicle control (control, *n* = 10; BPTES, *n* = 10). (**C**) Expression of *p16*^*INK4a*^ in the liver, lung, and kidney of young (8-week-old) and aged (80-week-old) mice treated with BPTES or vehicle control, as assessed by RT-qPCR (young, *n* = 13; control, *n* = 10; BPTES, *n* = 10). Fold changes were calculated using the ΔΔCT method relative to the young group. (**D**) Representative images of all mice taken before (day 0) and after (day 28) the completion of BPTES or vehicle administration (control, *n* = 10; BPTES, *n* = 10). Data are presented as mean ± s.e.m. Statistical significance was determined by the Mann–Whitney *U* test (**B**) and one-way ANOVA followed by Šídák’s multiple comparisons test (**C**). Day7, *p* = 0.36; Day14, **p = 0.0096; Day21, *p = 0.030; Day28, *p* = 0.066 (**B**). Young vs. Control: ****p < 0.0001 (Liver), *p* = 0.15 (Lung), **p = 0.0074 (Kidney); Young vs. BPTES: ***p = 0.0002 (Liver), **p = 0.0021 (Lung), *p = 0.029 (Kidney); Control vs. BPTES: *p* = 0.87 (Liver), *p* = 0.29 (Lung), *p* = 0.94 (Kidney) (**C**). n.s., not significant (*p* ≥ 0.05). Source data are available online for this figure.

**Figure 3:**
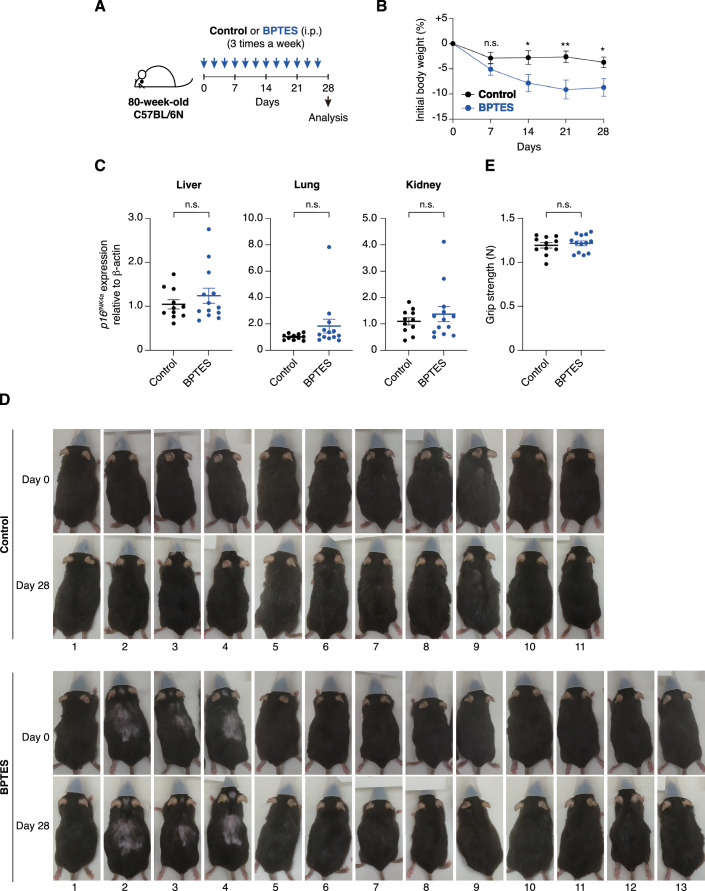
Blinded experiments failed to detect senolytic effects of BPTES in aged. (**A**) Timeline of the experimental procedure for BPTES administration in 80-week-old C57BL/6 N mice. (**B**) Body weight changes over time, shown relative to the starting weight, in mice treated with BPTES or vehicle control (control, *n* = 11; BPTES, *n* = 13). (**C**) Expression of *p16*^*INK4a*^ in the liver, lung, and kidney of 80-week-old mice treated with BPTES or vehicle control, assessed by RT-qPCR (control, *n* = 11; BPTES, *n* = 13). Fold changes were calculated using the ΔΔCT method relative to the vehicle control group. (**D**) Representative images of all mice before (day 0) and after (day 28) the completion of treatment with BPTES or vehicle control (control, *n* = 11; BPTES, *n* = 13). (**E**) Grip strength measurements in 80-week-old mice treated with BPTES or vehicle control (control, *n* = 11; BPTES, *n *= 13). Data are presented as mean ± s.e.m. Statistical significance was determined by the Mann–Whitney *U* test (**B**,** E**) and two-tailed unpaired *t*-test (**C**). Day7, *p* = 0.21; Day14, *p = 0.022; Day21, **p = 0.0088; Day28, *p = 0.035 (**B**). Liver, *p* = 0.36; Lung, *p* = 0.17; Kidney, *p* = 0.42 (**C**). *p* = 0.64 (**E**). n.s., not significant (*p* ≥ 0.05). Source data are available online for this figure.

To address this concern, we attempted to replicate the experiment previously reported by Johmura et al (2021), in which BPTES (0.25 mg per 20 g body weight in 200 μl) or vehicle control (200 μl of 10% DMSO in corn oil) was administered to 80-week-old male C57BL/6 mice three times per week for one month, in Hara’s laboratory (Fig. 2A,B). However, in contrast to the previous report (see Fig. S13A–C in Johmura et al, 2021), BPTES administration did not significantly reduce *p16*^*INK4a*^ expression in the liver, lung, or kidney, although the levels of *p16*^*INK4a*^ expression increased in these tissues with age (Fig. 2C). Furthermore, although the same group reported that BPTES administration to aged mice resulted in a visibly more youthful appearance, we were unable to confirm such changes in our study (Fig. 2D). Because aged mice exhibit high inter-individual variability, the resulting data dispersion poses a considerable risk of arbitrary or erroneous interpretation. To minimize potential bias, we designed experiments in which the analysts were blinded to the treatment allocation (BPTES or vehicle control). Specifically, in Oike’s laboratory (Kumamoto), 80-week-old male C57BL/6 mice were administered BPTES (0.25 mg per 20 g body weight in 200 μl) or vehicle control (200 μl of 10% DMSO in corn oil) three times per week for one month. Following euthanasia, organs were harvested, snap-frozen, and shipped to Hara’s laboratory with coded identifiers to maintain blinding. In Hara’s laboratory, RNA was extracted from these tissues and subjected to RT-qPCR analysis. All data were then transmitted to Oike’s laboratory, where the treatment codes were unblinded, and statistical analyses were performed (Fig. 3A–C). Consistent with our previous observations (Fig. 2), no significant differences in *p16*^*INK4a*^ expression in the liver, lung, or kidney tissues, or in external appearance, were observed between BPTES-treated and vehicle-treated mice (Fig. 3C,D). Furthermore, although BPTES treatment slightly reduced body weight, no significant differences were observed in physical parameters, such as grip strength, between the treatment groups (Fig. 3B,E). At present, the reasons underlying the discrepancy between our results and those reported by Johmura et al (2021) remain unclear. While our findings do not necessarily invalidate the data presented by Johmura et al (2021), they underscore the need for cautious interpretation of the senolytic effects of GLS1 inhibitors.

**Figure 4:**
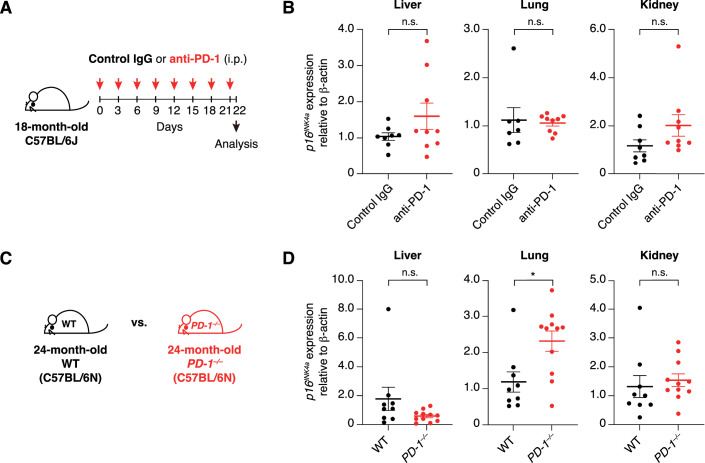
PD-1 blockade fails to exhibit senolytic effects in aged mice. (**A**) Timeline of the experimental procedure for anti-PD-1 antibody administration in 18-month-old C57BL/6 J mice. (**B**) *p16*^*INK4a*^ expression in the liver, lung, and kidney of 18-month-old C57BL/6 J mice treated with anti-PD-1 antibody or control IgG (control IgG, *n *= 7–8; anti-PD-1, *n *= 9), assessed by RT-qPCR. Fold changes were calculated using the ΔΔCT method relative to the control IgG group. (**C**) Schematic of the experimental design involving 24-month-old C57BL/6N-background wild-type (WT) and *PD-1*^*-/-*^ mice. (**D**) *p16*^*INK4a*^ expression in the liver, lung, and kidney of 24-month-old C57BL/6N-background WT and *PD-1*^*-/-*^ mice (WT, *n* = 9; *PD-1*^*-/-*^, *n* = 11), assessed by RT-qPCR. Fold changes were calculated using the ΔΔCT values relative to the WT group. Data are presented as mean ± s.e.m. Statistical significance was determined by two-tailed unpaired *t*-tests (**B**,** D**). Liver, *p* = 0.18; Lung, *p* = 0.80; Kidney, *p* = 0.14 (**B**). Liver, *p* = 0.12; Lung, *p = 0.011; Kidney, *p* = 0.60 (**D**). n.s., not significant (*p* ≥ 0.05). Source data are available online for this figure.

**Figure 5:**
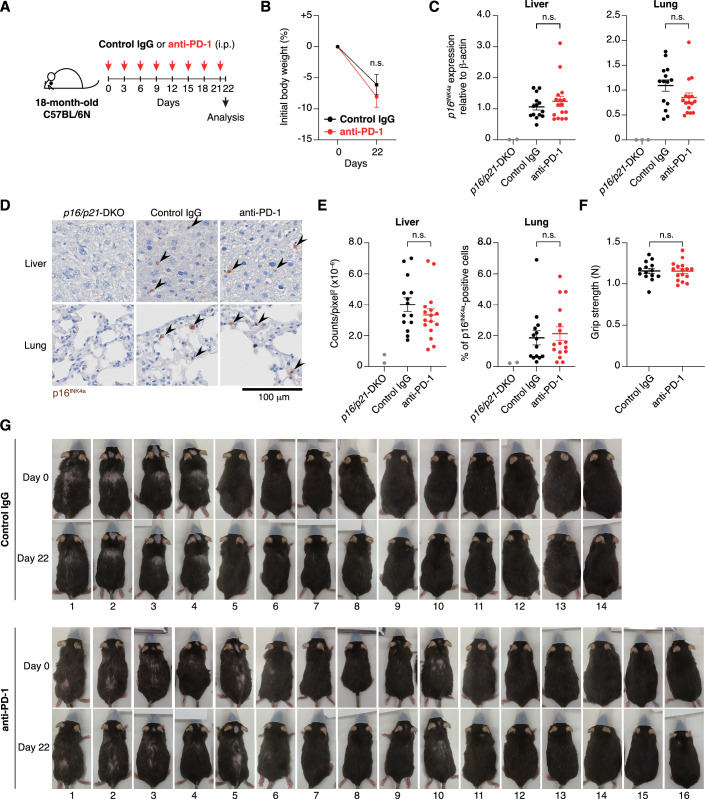
Blinded experiments failed to detect senolytic effects of PD-1 blockade in aged mice. (**A**) Timeline of the experimental procedure for anti-PD-1 antibody administration in 18-month-old C57BL/6 N mice. (**B**) Body weight changes over time, shown relative to the starting weight, in mice treated with anti-PD-1 antibody or control IgG (control IgG, *n* = 14; anti-PD-1, *n* = 16). (**C**–**E**) Expression of *p16*^*INK4a*^ in the liver and lung assessed by RT-qPCR (**C**), and representative images (**D**) and histological quantification of p16^INK4a^-positive cells (**E**) in liver and lung in 18-month-old C57BL/6 N mice treated with anti-PD-1 antibody or control IgG. For RT-qPCR analysis, fold changes were calculated using the ΔΔCT method relative to the control IgG group. *p16/p21*-DKO mice were included as a negative control (control IgG, *n* = 14; anti-PD-1, *n* = 16; *p16/p21*-DKO, *n* = 2–3). (**F**) Grip strength measurements in 18-month-old C57BL/6 N mice treated with anti-PD-1 antibody or control IgG (control IgG, *n* = 14; anti-PD-1, *n* = 16). (**G**) Representative images of all mice before (day 0) and after (day 22) the completion of treatment with anti-PD-1 antibody or control IgG (control IgG, *n* = 14; anti-PD-1, *n* = 16). Data are presented as mean ± s.e.m. Statistical significance was determined by the Mann–Whitney *U* test (**B**,** E**,** F**) and two-tailed unpaired *t*-test (**C**). The *p16/p21*-DKO samples were employed solely as negative controls and were excluded from statistical analyses (**C**–**E**). *p* = 0.45 (**B**). Liver, *p* = 0.37; Lung, *p* = 0.11 (**C**). Liver, *p* = 0.33; Lung, *p* = 0.70 (**E**). *p* = 0.89 (**F**). n.s., not significant (*p* ≥ 0.05). Source data are available online for this figure.

Given that the same group previously reported a reduction in senescent cells following anti-PD-1 antibody treatment in aged mice (Wang et al, 2022), we next sought to assess the reproducibility of this finding as well. To this end, 18-month-old male C57BL/6 mice were intraperitoneally injected with either isotype control IgG or anti-mouse PD-1 antibody (Chamoto et al, 2023) (250 μg per dose, 8 injections over a 3-week period) in Hara’s laboratory (Fig. 4A). One day after the final injection, the mice were euthanized, and *p16*^*INK4a*^ expression in the liver, lung, and kidney was quantified by RT-qPCR. However, in contrast to the previous report by Wang et al (see Fig. 4a, Extended Data Fig. 7c, d in 2022), the administration of the anti-mouse PD-1 antibody did not result in a statistically significant reduction in the levels of *p16*^*INK4a*^ gene expression in the liver, lung, or kidney (Fig. 4B). Furthermore, although we also examined *p16*^*INK4a*^ expression in aged (24-month-old) PD-1-deficient mice (Nishimura et al, 2001), no statistically significant differences were observed in the liver or kidney compared to age-matched wild-type controls (Fig. 4C,D). Interestingly, however, the *p16*^*INK4a*^ levels were unexpectedly increased, rather than decreased, in the lungs of PD-1-deficient mice (Fig. 4D). To ensure the validity of these results, we also designed blinded experiments in which the analysts were unaware of the treatment allocation (anti-PD-1 antibody or IgG control). Specifically, in Oike’s laboratory, 18-month-old male C57BL/6 mice were intraperitoneally injected with either isotype control IgG or anti-mouse PD-1 antibody (250 μg per dose, 8 injections over a 3-week period) (Fig. 5A,B). After euthanasia, liver and lung tissues were harvested, immediately snap-frozen, and shipped to Hara’s laboratory with coded identifiers to maintain blinding. In Hara’s laboratory, these tissues were subjected to RT-qPCR or immunohistochemical analysis using an anti-p16^INK4a^ antibody. For the immunohistochemical analysis, image data were analyzed using computational methods in Matsuda’s laboratory (Kyoto). All data were then transmitted to Oike’s laboratory, where the treatment codes were unblinded, and statistical analyses were conducted. Consistent with our previous observations (Fig. 4), we detected no statistically significant differences in *p16*^*INK4a*^ RNA or protein levels in liver and lung tissues between the anti-PD-1 antibody-treated and control IgG-treated mice (Fig. 5C–E). Furthermore, although the anti-PD-1 antibody treatment slightly reduced the body weight, no significant differences were observed between the treatment groups in external appearance or physical parameters, such as grip strength (Fig. 5B,F,G). This is broadly consistent with a recent report indicating that anti-PD-1 antibody administration failed to reduce the number of p16^INK4a^-positive cells in inflamed lung tissue in mice (Majewska et al, 2024). Together, although our findings do not entirely refute those reported by Wang et al (2022), they highlight the need for cautious interpretation of the senolytic effects of anti-PD-1 antibodies.

At present, the reasons underlying the discrepancy between our results and those reported by Johmura et al (2021) and Wang et al (2022) remain unclear. It is well recognized that biological experiments are often difficult to reproduce, as even subtle differences in experimental conditions can alter outcomes. In this study, however, the BPTES treatment experiments in aged mice were conducted under the guidance of Dr. Sugimoto, who was directly responsible for the corresponding experiments in Johmura et al (2021) and is also a co-author of the present study. We therefore consider it unlikely that methodological discrepancies played a major role in this case. By contrast, aged mice are known to exhibit pronounced inter-individual variability (Hamieh et al, 2021), which may have contributed to the differences between the data of Johmura et al (2021) and Wang et al (2022) and our own. Senolytic drugs, however, are ultimately intended for use in humans, who display even greater inter-individual variability than laboratory mice. Therefore, experimental designs should aim to yield robust results that minimize the influence of both inter-individual variability and experimenter-dependent variability. In this regard, cross-laboratory, blinded studies, such as the one conducted here, represent a promising strategy.

Another consideration is age. Eighty-week-old and 18-month-old 57BL/6 mice cannot be regarded as genuinely old. We nevertheless selected this age to ensure comparability with Johmura et al (2021) and Wang et al (2022). Although Johmura et al (2021) also examined 100-week-old mice, their analyses were limited to adipose tissue, where abundant macrophages complicate interpretation due to *p16*^*INK4a*^ expression and SA-β-gal activity independent of senescence (Hall et al, 2017). To avoid these confounders, we focused on lungs, liver, and kidney in 80-week-old mice. Consistent with Johmura et al (2021), we observed increased* p16*^*INK4a*^ expression in these tissues with age (Fig. 2C), although no reduction was observed after BPTES treatment.

An important point to note is that Wang et al (2022) did not directly assess endogenous *p16*^*INK4a*^ expression, but instead quantified tdTomato reporter–positive cells using the *p16-CreERT2-tdTomato* mouse model (Omori et al, 2020). Notably, this system is designed for lineage tracing rather than real-time monitoring of *p16*^*INK4a*^ expression. Therefore, tdTomato positivity reflects cells that have previously activated the *p16*^*INK4a*^ promoter, and may not accurately represent the current endogenous *p16*^*INK4a*^ expression levels. To investigate this possibility, we reanalyzed the single-cell RNA sequencing (scRNA-seq) data from the livers and kidneys of *p16-CreERT2-tdTomato* mice previously reported by Omori et al (2020). However, we could not detect any expression of the *Cdkn2a* gene, which encodes *p16*^*INK4a*^. This raises uncertainty as to whether tdTomato expression reliably reflects endogenous *p16*^*INK4a*^ expression. We therefore emphasize the importance of directly assessing the endogenous *p16*^*INK4a*^ expression to ensure the accurate interpretation of experimental results. Furthermore, *p16*^*INK4a*^ expression alone cannot be regarded as a definitive marker of aging in mice (Hall et al, 2017). Nevertheless, our study was not designed to establish such a marker, but rather to test the reproducibility of the key findings reported by Johmura et al (2021) and Wang et al (2022), which suggested the strong senolytic activities of BPTES and anti-PD1 antibody—claims that have attracted substantial public attention and hold profound societal consequences. Given this context, we deliberately focused on reproducing the datasets most central to those reports. In both studies, *p16*^*INK4a*^ expression was employed as a key marker of cellular senescence, while grip strength served as an indicator of organismal aging (Johmura et al, 2021; Wang et al, 2022). Moreover, the same groups have emphasized through mass media that treatments with these drugs induce an apparent rejuvenation in the physical appearance of mice. Because such photographic evidence can strongly influence public perception, we have included photographs of all mice before and after treatment with either BPTES or anti-PD1 antibody (Figs. 2D, 3D, and 5G).

Why is it challenging to confirm the reproducibility of experimental data in senolytic research (Harrison et al, 2024; Kovacovicova et al, 2018)? One possible explanation is that inter-individual variability in mice increases with age (Hamieh et al, 2021), in parallel with the heterogeneity of senescent cells (Tao et al, 2024; Wechter et al, 2023). To address this, we recommend increasing the number of animals to account for such variability and conducting blinded experiments in collaboration with multiple laboratories to minimize researcher bias (i.e., ensuring that experimenters are unaware of treatment allocation). Finally, we emphasize that our intention is not to discredit previous work by any specific group, but rather to provide an independent and rigorous validation, which we believe is valuable for the field as senolytic therapies advance toward clinical applications.

## Methods

**Table:** Reagents and tools table

Reagent/Resource	Reference or Source	Identifier or Catalog Number
**Experimental models**		
TIG-1	JCRB Cell Bank	JCRB0501
TIG-3	JCRB Cell Bank	JCRB0506
IMR-90	JCRB Cell Bank	JCRB9054
C57BL/6 J (*M. musculus*)	CLEA Japan, the National BioResource Project of the Ministry of Education, Culture, Sports, Science and Technology in Japan	C57BL/6JJcl
C57BL/6 N (*M. musculus*)	CLEA Japan	C57BL/6NJcl
*PD-1*-deficient mice (*M. musculus*)	Nishimura et al, 2001	
*p16/p21* double knockout mice (*M. musculus*)	Kawamoto et al, 2023	
**Antibodies**		
Mouse anti-β-actin	Sigma-Aldrich	A5316
Mouse anti-β-actin	Sigma-Aldrich	A4700
Rabbit anti-Lamin B1	Abcam	ab16048
Mouse anti-p16^INK4a^	Santa Cruz	sc-56330
Mouse anti-p16^INK4a^	IBL	11104
Rabbit anti-p21^Waf1/Cip1^	Cell Signaling Technology	2947
HRP-conjugated anti-rabbit or mouse IgG	Cell Signaling Technology	7074, 7076
GoInVivo™ Purified Rat IgG2a, κ Isotype Ctrl Antibody	BioLegend	400563
GoInVivo™ Purified anti-mouse CD279 (PD-1) Antibody	BioLegend	135234
Rabbit anti-p16^INK4a^	Abcam	ab211542
**Oligonucleotides and other sequence-based reagents**		
Mouse *Actb* primers	Kawamoto et al, 2023	Methods and Protocols
Mouse *p16*^*INK4a*^ primers	Kawamoto et al, 2023	Methods and Protocols
**Chemicals, Enzymes and other reagents**		
DMEM	Nacalai tesque	08458-16
MEM	Nacalai tesque	21442-25
Fetal bovine serum	MP Biomedicals	2917345H
Fetal bovine serum	Sigma-Aldrich	173012
MEM Nonessential Amino Acid Solution	Sigma-Aldrich	06344-56
Penicillin-streptomycin-glutamine	Sigma-Aldrich	P4333
Penicillin-streptomycin-glutamine	Gibco	C10378-016
Doxorubicin	FUJIFILM Wako Pure Chemical Corporation	046-21523
Etoposide	Sigma-Aldrich	341205
BPTES	Sigma-Aldrich	SML0601
BPTES	Cayman	19284
Protease inhibitor cocktail	Nacalai Tesque	25955-11
Protein Quantification Assay	Takara Bio	740967.250
PVDF membrane	EMD Millipore	IPVH00010
Amersham ECL Prime or Select reagent	GE Healthcare	RPN2236 or RPN2235
Alexa Fluor 488 Click-iT EdU Imaging Kit	Thermo Fisher Scientific	C10637
DAPI	Dojindo	340-07971
FITC-conjugated annexin V	Thermo Fisher Scientific	A13199
Bouin’s fixative	Muto Pure Chemicals	33142
PathoClean	FUJIFILM Wako Pure Chemical Corporation	161-28321
ImmPRESS HRP horse anti-rabbit IgG polymer detection kit	Vector Laboratories	MP-7401
ImmPACT DAB substrate kit	Vector Laboratories	SK-4105
Hematoxylin	Muto Pure Chemicals	30002
PARAmount	FALMA	308-400
RNeasy Mini Kit	QIAGEN	74106
PrimeScript RT Reagent Kit with gDNA Eraser	Takara Bio	RR047A
TB Green Premix Ex Taq II	Takara Bio	RR820A
**Software**		
Fiji	Schindelin et al, 2012	
MATLAB R2024a	The MathWorks, Inc.	
Cellpose	Stringer et al, 2021	
Prism v10.4.0	GraphPad software	
**Other**		
All-in-One Fluorescence Microscope	Keyence	BZ-X710
Amersham ImageQuant 800 system	Cytiva	
Grip strength meters	Columbus Instruments	
Microtome	Leica Biosystems	
SLIDEVIEW VS200 Universal Whole Slide Imaging Scanner	Evident	
Thermal Cycler Dice Real Time System III	Takara Bio	

### Cell culture

Normal human diploid fibroblasts (HDFs), including TIG-1 (JCRB0501), TIG-3 (JCRB0506), and IMR-90 (JCRB9054), were obtained from the Japanese Collection of Research Bioresources (JCRB) Cell Bank. TIG-3 and IMR-90 cells were cultured in Dulbecco’s modified Eagle’s medium (DMEM; 08458-16, Nacalai Tesque) supplemented with 10% fetal bovine serum (FBS; 2917345H, MP Biomedicals, or 173012, Sigma-Aldrich) and 100 U/ml penicillin-streptomycin (P4333; Sigma-Aldrich). TIG-1 cells were cultured in Modified Eagle’s Medium (MEM; 21442-25, Nacalai Tesque) supplemented with 10% FBS, MEM Nonessential Amino Acid Solution (06344-56, Sigma-Aldrich), and penicillin-streptomycin-glutamine (P4333, Sigma-Aldrich, or C10378-016, Gibco). Cellular senescence was induced by treating cells with 250 ng/ml doxorubicin (046-21523, FUJIFILM Wako Pure Chemical Corporation) for 10 days, or 100 μM etoposide (341205, Sigma-Aldrich) for 2 days followed by a 10-day recovery period in normal culture medium. The pH of the culture medium was adjusted as needed using NaOH or HCl. To evaluate the senolytic activity of BPTES (SML0601, Sigma-Aldrich, or 19284, Cayman), 1.5 × 10⁵ cells were seeded in 12-well plates with grids, and cell numbers at fixed positions were recorded daily using a BZ-X710 All-in-One Fluorescence Microscope (Keyence). Relative cell number was calculated as follows: the average cell count on day 0 was defined as 100%, and the number of cells at each time point was expressed as a percentage relative to the day 0 value.

### Western blotting analysis

Cells were lysed in RIPA buffer supplemented with 1% protease inhibitor cocktail (25955-11, Nacalai Tesque). Protein concentrations were determined using the Protein Quantification Assay (740967.250, Takara Bio). Samples were denatured in Laemmli sample buffer by heating at 95 °C for 5 min. Proteins were separated by SDS–polyacrylamide gel electrophoresis and transferred onto PVDF membranes (IPVH00010, EMD Millipore). Membranes were blocked with 5% non-fat milk and incubated with the following primary antibodies: β-actin (1:2000; A5316, Sigma-Aldrich, 1:1000; A4700, Sigma-Aldrich), Lamin B1 (1:1000; ab16048, Abcam), p16^INK4a^ (1:1000; sc-56330, Santa Cruz, 1:1000; 11104, IBL), and p21^Waf1/Cip1^ (1:1000; 2947, Cell Signaling Technology). After washing, membranes were incubated with HRP-conjugated secondary antibodies (1:2000; 7074 and 7076, Cell Signaling Technology) and developed using Amersham ECL Prime or Select reagents (RPN2236 or RPN2235, GE Healthcare). Chemiluminescent signals were detected using the Amersham ImageQuant 800 system (Cytiva). Uncropped and unprocessed blot images are provided in the Source Data file.

### EdU incorporation assay

EdU incorporation analysis was performed using the Alexa Fluor 555 Click-iT EdU Imaging Kit (C10637, Thermo Fisher Scientific), with the following modifications to the manufacturer’s protocol. Cells were incubated with 50 µM EdU for 7 days prior to fixation. Following fixation, two sequential 1-h click reactions were performed using freshly prepared reaction solutions. Coverslips were then washed three times with 3% BSA in PBS. Nuclei were counterstained with Hoechst33342 (H342, Dojindo). EdU-positive signals were acquired using an all-in-one fluorescence microscope (BZ-710, Keyence). Quantification was performed using Fiji (Schindelin et al, 2012).

### Apoptosis assay

Apoptotic cells were detected using annexin V staining or TUNEL staining. For annexin V staining, cells were stained with FITC-conjugated annexin V (A13199, Thermo Fisher Scientific) in binding buffer (10 mM HEPES, 140 mM NaCl, and 2.5 mM CaCl₂, pH 7.4) for 15 min. Nuclei were counterstained with DAPI (340-07971, Dojindo). For TUNEL staining, 4% PFA-fixed cells were processed using the One-step TUNEL In Situ Apoptosis Kit (Red, Elab Fluor 555, E-CA-A325, Elabscience) according to the manufacturer’s instructions. Nuclei were counterstained with DAPI (340-07971, Dojindo). Images were acquired using an all-in-one fluorescence microscope (BZ-710, Keyence), and quantification was performed using Fiji (ImageJ).

### Mice

C57BL/6 J or C57BL/6 N male mice (8-week-old or 80-week-old, and 18-month-old) were purchased from CLEA Japan. Some of the aged mice were provided by the National BioResource Project (NBRP) of the Ministry of Education, Culture, Sports, Science and Technology in Japan (MEXT) through the Foundation for Biomedical Research and Innovation at Kobe, and by the Japan Agency for Medical Research and Development (AMED) under grant number JP24gm1710001h0003. *PD-1* knockout (*PD-1⁻/⁻*) (Nishimura et al, 2001), *p16/p21* double knockout (DKO) (Kawamoto et al, 2023), and wild-type (WT) mice on a C57BL/6 background were bred and maintained under specific pathogen-free (SPF) conditions at the RIKEN Center for Integrative Medical Sciences (IMS) (AEY2024-009), the Research Institute for Microbial Diseases (RIMD) at the University of Osaka (Biken-AP-R07-05-0), and Kumamoto University (A2024-123). All mice were housed in a temperature- (23–25 °C) and humidity-controlled environment on a 12-h light/dark cycle, and were fed standard chow (CE-2; CLEA Japan) sterilized by 20 kGy gamma irradiation. All animal experiments were conducted in accordance with protocols approved by the Animal Research Committees of RIKEN IMS, RIMD at the University of Osaka, or Kumamoto University. Mice were euthanized using carbon dioxide, and all efforts were made to minimize suffering. To minimize potential confounding factors, mice that exhibited tumor development were excluded from all downstream analyses.

### BPTES administration

BPTES administration was performed as previously described in Johmura et al (2021). Male C57BL/6 J or C57BL/6 N mice (8 or 80 weeks old) were intraperitoneally injected with BPTES (SML0601; Sigma-Aldrich) at a dose of 0.25 mg per 20 g body weight in 200 μl volume, or with a vehicle control (200 μl of 10% DMSO in corn oil), three times per week for one month. In certain experiments, randomization of samples was performed, and the procedures were carried out in a blinded fashion by different experimental facilities and personnel.

### Anti-PD-1 antibody administration

Anti-PD-1 antibody administration was performed as previously described in Wang et al (2022). Eighteen-month-old male C57BL/6 J or C57BL/6 N mice were intraperitoneally injected with 250 μg of either isotype control IgG (clone RTK2758; 400563, BioLegend) or anti-mouse PD-1 antibody (clone 29F.1A12; 135234, BioLegend) every 3 days for 3 weeks. Mice were euthanized one day after the final injection. In certain experiments, randomization of samples was performed, and the procedures were carried out in a blinded fashion by different experimental facilities and personnel.

### Measurement of grip strength

Forelimb grip strength (in newtons, N) was measured using a grip strength meter (Columbus Instruments, Columbus, OH). Each mouse was gently held by the base of the tail and allowed to grasp a horizontal metal bar with its forepaws. The mouse was then pulled backward in a horizontal plane until it released the bar. The peak force exerted before release was recorded as the grip strength. Each mouse was tested three consecutive times, and the highest value among the three trials was used for analysis. All measurements were performed by the same experimenter.

### Immunohistochemistry

Tissue specimens were fixed in Bouin’s fixative (33142, Muto Pure Chemicals) for 2 h at room temperature, soaked overnight in 70% ethanol, embedded in paraffin, and sectioned at a thickness of 5 μm using a microtome (Leica Biosystems). The sections were deparaffinized in PathoClean (161-28321, FUJIFILM Wako Pure Chemical Corporation), rehydrated, and subjected to antigen retrieval by microwave heating in 10 mM citrate buffer (pH 6.0) for 20 min. After blocking with 5% horse serum, the sections were treated with 2% hydrogen peroxide for 10 min to inactivate endogenous peroxidase activity. Anti-mouse p16^INK4a^ (1:1000; ab211542, Abcam) was used as the primary antibody (Doolittle et al, 2023; Kawamoto et al, 2023). Signal detection was performed using the ImmPRESS HRP horse anti-rabbit IgG polymer detection kit (MP-7401, Vector Laboratories) and the ImmPACT DAB substrate kit (SK-4105, Vector Laboratories). Finally, the sections were counterstained with hematoxylin (30002, Muto Pure Chemicals), dehydrated, and mounted with PARAmount (308-400, FALMA).

### Image processing and analysis for p16^INK4a^-positive cell quantification

Tissue sections were digitally scanned using SLIDEVIEW VS200 Universal Whole Slide Imaging Scanner (Evident) equipped with a 20× objective lens. The resulting images were converted to TIFF format and processed using ImageJ Fiji (Schindelin et al, 2012). Immunohistochemical staining was quantified using a custom macro that performed color deconvolution to separate the images into three components: hematoxylin (nuclei), DAB (positive immunostaining), and residual staining. Each separated channel was saved as an individual TIFF file. For lung tissue samples, a MATLAB-based pipeline incorporating Cellpose was used (Stringer et al, 2021; MATLAB R2024a, The MathWorks, Inc.). The workflow involved processing each image with Cellpose, and quantifying total cells and DAB-positive cells. For liver tissue samples, we used a custom ImageJ macro. First, hematoxylin-stained images were used to define tissue boundaries. These images were subjected to Gaussian blurring, followed by binary thresholding to generate tissue masks. DAB-positive cell counting was conducted using the “Find Maxima” function of Fiji.

### Quantitative real-time PCR analysis

Total RNA was extracted from tissues using the RNeasy Mini Kit (74106, QIAGEN) according to the manufacturer’s instructions. A total of 1000 ng of RNA was used to synthesize cDNA with the PrimeScript RT Reagent Kit with gDNA Eraser (RR047A, Takara Bio). Quantitative real-time PCR was performed using the Thermal Cycler Dice Real Time System III (Takara Bio) and the TB Green Premix Ex Taq II (RR820A, Takara Bio). The mRNA expression level of each gene was normalized to β-actin (*Actb*) expression. Relative expression levels were calculated using the ∆∆CT method. The specific primers used were as follows:

- *Actb*: 5′-GATGACCCAGATCATGTTTGA-3′ and 5′-GGAGAGCATAGCCCTCGTAG-3′
- *p16*^*INK4a*^: 5′-GAACTCTTTCGGTCGTACCC-3′ and 5′-CGAATCTGCACCGTAGTTGA-3′

### Statistical analysis

The graphs or plots are presented as mean ± SD (standard deviation) or mean ± SEM (standard error of the mean) with sample size ≥3 and indicated in the figure legend. The datasets were not tested for normality. All data were visualized and analyzed using Prism (version 10.4.0; GraphPad Software). Statistical significance was assessed using the one-way ANOVA with Dunnett’s test (Fig. 1A,C), Mann–Whitney U test (Figs. 2B, 3B,E and 5B,E,G), one-way ANOVA followed by Šídák’s multiple comparisons test (Fig. 2C), or a two-tailed unpaired *t*-test (Figs. 3C, 4B,D, and 5C). Sample size estimation was not performed.

## Supplementary information

Peer Review File

Source data Fig. 1

Source data Fig. 2

Source data Fig. 3

Source data Fig. 4

Source data Fig. 5

Figure EV1 Source Data

Figure EV2 Source Data

Expanded View Figures

## Data Availability

This study includes no data deposited in external repositories. The source data of this paper are collected in the following database record: biostudies:S-SCDT-10_1038-S44319-026-00740-5.

## References

[CR1] Acosta JC, O’Loghlen A, Banito A, Guijarro MV, Augert A, Raguz S, Fumagalli M, Da Costa M, Brown C, Popov N et al (2008) Chemokine signaling via the CXCR2 receptor reinforces senescence. Cell 133:1006–1018 18555777 10.1016/j.cell.2008.03.038

[CR2] Amor C, Feucht J, Leibold J, Ho YJ, Zhu C, Alonso-Curbelo D, Mansilla-Soto J, Boyer JA, Li X, Giavridis T et al (2020) Senolytic CAR T cells reverse senescence-associated pathologies. Nature 583:127–132 32555459 10.1038/s41586-020-2403-9 PMC7583560

[CR3] Baker DJ, Childs BG, Durik M, Wijers ME, Sieben CJ, Zhong J, Saltness RA, Jeganathan KB, Verzosa GC, Pezeshki A et al (2016) Naturally occurring p16^Ink4a^-positive cells shorten healthy lifespan. Nature 530:184–189 26840489 10.1038/nature16932 PMC4845101

[CR4] Baker DJ, Wijshake T, Tchkonia T, LeBrasseur NK, Childs BG, van de Sluis B, Kirkland JL, van Deursen JM (2011) Clearance of p16^Ink4a^-positive senescent cells delays ageing-associated disorders. Nature 479:232–236 22048312 10.1038/nature10600 PMC3468323

[CR5] Campisi J, d’Adda di Fagagna F (2007) Cellular senescence: when bad things happen to good cells. Nat Rev Mol Cell Biol 8:729–740 17667954 10.1038/nrm2233

[CR6] Chaib S, Tchkonia T, Kirkland JL (2022) Cellular senescence and senolytics: the path to the clinic. Nat Med 28:1556–1568 35953721 10.1038/s41591-022-01923-y PMC9599677

[CR7] Chamoto K, Yaguchi T, Tajima M, Honjo T (2023) Insights from a 30-year journey: function, regulation and therapeutic modulation of PD1. Nat Rev Immunol 23:682–695 37185300 10.1038/s41577-023-00867-9

[CR8] Chan ASL, Narita M (2019) Short-term gain, long-term pain: the senescence life cycle and cancer. Genes Dev 33:127–143 30709901 10.1101/gad.320937.118 PMC6362810

[CR9] Collado M, Serrano M (2010) Senescence in tumours: evidence from mice and humans. Nat Rev Cancer 10:51–57 20029423 10.1038/nrc2772 PMC3672965

[CR10] Coppe JP, Patil CK, Rodier F, Sun Y, Munoz DP, Goldstein J, Nelson PS, Desprez PY, Campisi J (2008) Senescence-associated secretory phenotypes reveal cell-nonautonomous functions of oncogenic RAS and the p53 tumor suppressor. PLoS Biol 6:2853–2868 19053174 10.1371/journal.pbio.0060301 PMC2592359

[CR12] Doolittle ML, Saul D, Kaur J, Rowsey JL, Vos SJ, Pavelko KD, Farr JN, Monroe DG, Khosla S (2023) Multiparametric senescent cell phenotyping reveals targets of senolytic therapy in the aged murine skeleton. Nat Commun 14:4587 37524694 10.1038/s41467-023-40393-9 PMC10390564

[CR13] Gorgoulis V, Adams PD, Alimonti A, Bennett DC, Bischof O, Bishop C, Campisi J, Collado M, Evangelou K, Ferbeyre G et al (2019) Cellular senescence: defining a path forward. Cell 179:813–827 31675495 10.1016/j.cell.2019.10.005

[CR14] Grosse L, Wagner N, Emelyanov A, Molina C, Lacas-Gervais S, Wagner KD, Bulavin DV (2020) Defined p16^High^ senescent cell types are indispensable for mouse healthspan. Cell Metab 32:87–99.e86 32485135 10.1016/j.cmet.2020.05.002

[CR15] Hall BM, Balan V, Gleiberman AS, Strom E, Krasnov P, Virtuoso LP, Rydkina E, Vujcic S, Balan K, Gitlin II et al (2017) p16^Ink4a^ and senescence-associated beta-galactosidase can be induced in macrophages as part of a reversible response to physiological stimuli. Aging 9:1867–1884 28768895 10.18632/aging.101268 PMC5611982

[CR16] Hamieh AM, Camperos E, Hernier AM, Castagné V (2021) C57BL/6 mice as a preclinical model to study age-related cognitive deficits: Executive functions impairment and inter-individual differences. Brain Res 1751:147–173 10.1016/j.brainres.2020.147173 33148432

[CR17] Harrison DE, Strong R, Reifsnyder P, Rosenthal N, Korstanje R, Fernandez E, Flurkey K, Ginsburg BC, Murrell MD, Javors MA et al (2024) Astaxanthin and meclizine extend lifespan in UM-HET3 male mice; fisetin, SG1002 (hydrogen sulfide donor), dimethyl fumarate, mycophenolic acid, and 4-phenylbutyrate do not significantly affect lifespan in either sex at the doses and schedules used. GeroScience 46:795–816 38041783 10.1007/s11357-023-01011-0 PMC10828146

[CR18] He S, Sharpless NE (2017) Senescence in health and disease. Cell 169:1000–1011 28575665 10.1016/j.cell.2017.05.015 PMC5643029

[CR19] Johmura Y, Yamanaka T, Omori S, Wang TW, Sugiura Y, Matsumoto M, Suzuki N, Kumamoto S, Yamaguchi K, Hatakeyama S et al (2021) Senolysis by glutaminolysis inhibition ameliorates various age-associated disorders. Science 371:265–270 33446552 10.1126/science.abb5916

[CR20] Kang TW, Yevsa T, Woller N, Hoenicke L, Wuestefeld T, Dauch D, Hohmeyer A, Gereke M, Rudalska R, Potapova A et al (2011) Senescence surveillance of pre-malignant hepatocytes limits liver cancer development. Nature 479:547–551 22080947 10.1038/nature10599

[CR21] Kawamoto S, Uemura K, Hori N, Takayasu L, Konishi Y, Katoh K, Matsumoto T, Suzuki M, Sakai Y, Matsudaira T et al (2023) Bacterial induction of B cell senescence promotes age-related changes in the gut microbiota. Nat Cell Biol 25:865–876 37169880 10.1038/s41556-023-01145-5

[CR22] Kovacovicova K, Skolnaja M, Heinmaa M, Mistrik M, Pata P, Pata I, Bartek J, Vinciguerra M (2018) Senolytic cocktail dasatinib+quercetin (D+Q) does not enhance the efficacy of senescence-inducing chemotherapy in liver cancer. Front Oncol 8:459 30425964 10.3389/fonc.2018.00459 PMC6218402

[CR23] Krishnamurthy J, Torrice C, Ramsey MR, Kovalev GI, Al-Regaiey K, Su L, Sharpless NE (2004) *Ink4a/Arf* expression is a biomarker of aging. J Clin Invest 114:1299–1307 15520862 10.1172/JCI22475 PMC524230

[CR24] Krizhanovsky V, Yon M, Dickins RA, Hearn S, Simon J, Miething C, Yee H, Zender L, Lowe SW (2008) Senescence of activated stellate cells limits liver fibrosis. Cell 134:657–667 18724938 10.1016/j.cell.2008.06.049 PMC3073300

[CR25] Kuilman T, Michaloglou C, Vredeveld LC, Douma S, van Doorn R, Desmet CJ, Aarden LA, Mooi WJ, Peeper DS (2008) Oncogene-induced senescence relayed by an interleukin-dependent inflammatory network. Cell 133:1019–1031 18555778 10.1016/j.cell.2008.03.039

[CR26] Lee S, Schmitt CA (2019) The dynamic nature of senescence in cancer. Nat Cell Biol 21:94–101 30602768 10.1038/s41556-018-0249-2

[CR27] Majewska J, Agrawal A, Mayo A, Roitman L, Chatterjee R, Sekeresova Kralova J, Landsberger T, Katzenelenbogen Y, Meir-Salame T, Hagai E et al (2024) p16-dependent increase of PD-L1 stability regulates immunosurveillance of senescent cells. Nat Cell Biol 26:1336–1345 39103548 10.1038/s41556-024-01465-0 PMC11321988

[CR28] Naylor RM, Baker DJ, van Deursen JM (2013) Senescent cells: a novel therapeutic target for aging and age-related diseases. Clin Pharm Ther 93:105–116 10.1038/clpt.2012.193 PMC4051295 23212104

[CR29] Nishimura H, Okazaki T, Tanaka Y, Nakatani K, Hara M, Matsumori A, Sasayama S, Mizoguchi A, Hiai H, Minato N et al (2001) Autoimmune dilated cardiomyopathy in PD-1 receptor-deficient mice. Science 291:319–322 11209085 10.1126/science.291.5502.319

[CR30] Omori S, Wang TW, Johmura Y, Kanai T, Nakano Y, Kido T, Susaki EA, Nakajima T, Shichino S, Ueha S et al (2020) Generation of a p16 reporter mouse and its use to characterize and target p16^high^ cells in vivo. Cell Metab 32:814–828.e816 32949498 10.1016/j.cmet.2020.09.006

[CR31] Power H, Valtchev P, Dehghani F, Schindeler A (2023) Strategies for senolytic drug discovery. Aging Cell 22:e13948 37548098 10.1111/acel.13948 PMC10577556

[CR32] Reyes NS, Krasilnikov M, Allen NC, Lee JY, Hyams B, Zhou M, Ravishankar S, Cassandras M, Wang C, Khan I et al (2022) Sentinel p16^INK4a^+ cells in the basement membrane form a reparative niche in the lung. Science 378:192–201 36227993 10.1126/science.abf3326 PMC10621323

[CR33] Schindelin J, Arganda-Carreras I, Frise E, Kaynig V, Longair M, Pietzsch T, Preibisch S, Rueden C, Saalfeld S, Schmid B et al (2012) Fiji: an open-source platform for biological-image analysis. Nat Methods 9:676–682 22743772 10.1038/nmeth.2019 PMC3855844

[CR34] Serrano M, Blasco MA (2001) Putting the stress on senescence. Curr Opin Cell Biol 13:748–753 11698192 10.1016/s0955-0674(00)00278-7

[CR35] Stringer C, Wang T, Michaelos M, Pachitariu M (2021) Cellpose: a generalist algorithm for cellular segmentation. Nat Methods 18:100–106 33318659 10.1038/s41592-020-01018-x

[CR36] Suda M, Shimizu I, Katsuumi G, Yoshida Y, Hayashi Y, Ikegami R, Matsumoto N, Yoshida Y, Mikawa R, Katayama A et al (2021) Senolytic vaccination improves normal and pathological age-related phenotypes and increases lifespan in progeroid mice. Nat Aging 1:1117–1126 37117524 10.1038/s43587-021-00151-2

[CR37] Tao W, Yu Z, Han JJ (2024) Single-cell senescence identification reveals senescence heterogeneity, trajectory, and modulators. Cell Metab 36:1126–1143.e1125 38604170 10.1016/j.cmet.2024.03.009

[CR38] van Deursen JM (2019) Senolytic therapies for healthy longevity. Science 364:636–637 31097655 10.1126/science.aaw1299 PMC6816502

[CR39] Wakita M, Ito K, Fuji K, Sakamoto D, Mikawa T, Sugawara S, Zhou X, Park JH, Miyagawa H, Motooka D et al (2026) Comparative analysis of senolytic drugs reveals mitochondrial determinants of efficacy and resistance. Nat Aging 6: 316-328 10.1038/s43587-025-01057-z PMC12920139 41611832

[CR11] Wang L, Lankhorst L, Bernards R (2022) Exploiting senescence for the treatment of cancer. Nat Rev Cancer 22:340–355. 10.1038/s41568-022-00450-9 35241831

[CR40] Wang TW, Johmura Y, Suzuki N, Omori S, Migita T, Yamaguchi K, Hatakeyama S, Yamazaki S, Shimizu E, Imoto S et al (2022) Blocking PD-L1-PD-1 improves senescence surveillance and ageing phenotypes. Nature 611:358–364 36323784 10.1038/s41586-022-05388-4

[CR41] Watanabe S, Kawamoto S, Ohtani N, Hara E (2017) Impact of senescence-associated secretory phenotype and its potential as a therapeutic target for senescence-associated diseases. Cancer Sci 108:563–569 28165648 10.1111/cas.13184 PMC5406532

[CR42] Wechter N, Rossi M, Anerillas C, Tsitsipatis D, Piao Y, Fan J, Martindale JL, De S, Mazan-Mamczarz K, Gorospe M (2023) Single-cell transcriptomic analysis uncovers diverse and dynamic senescent cell populations. Aging 15:2824–2851 37086265 10.18632/aging.204666 PMC10188353

[CR43] Yamakoshi K, Takahashi A, Hirota F, Nakayama R, Ishimaru N, Kubo Y, Mann DJ, Ohmura M, Hirao A, Saya H et al (2009) Real-time in vivo imaging of p16^Ink4a^ reveals cross talk with p53. J Cell Biol 186:393–407 19667129 10.1083/jcb.200904105 PMC2728398

[CR44] Yoshida S, Nakagami H, Hayashi H, Ikeda Y, Sun J, Tenma A, Tomioka H, Kawano T, Shimamura M, Morishita R et al (2020) The CD153 vaccine is a senotherapeutic option for preventing the accumulation of senescent T cells in mice. Nat Commun 11:2482 32424156 10.1038/s41467-020-16347-w PMC7235045

[CR45] Zhao H, Liu Z, Chen H, Han M, Zhang M, Liu K, Jin H, Liu X, Shi M, Pu W et al (2024) Identifying specific functional roles for senescence across cell types. Cell 187:7314–7334.e7321 39368477 10.1016/j.cell.2024.09.021

