# Supplementary figures and images for: Reevaluating the senolytic activity of a GLS1 inhibitor and an anti-PD-1 antibody: toward greater reproducibility and methodological rigor
Source: EMBO Rep. 2026 Apr 3;27(9):2201–17. doi: 10.1038/s44319-026-00740-5 (PMC13172396; doi:10.1038/s44319-026-00740-5)

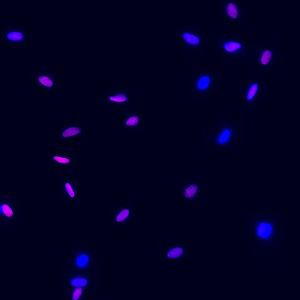

Supplement: Supplementary file 2 — Source data Fig. 1 [file 44319_2026_740_MOESM2_ESM.zip › Figure 1/1A/1A_IF_Control.tif]

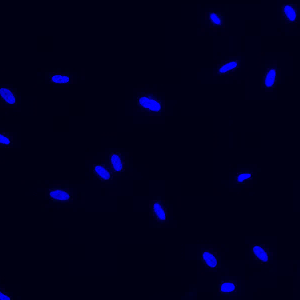

Supplement: Supplementary file 2 — Source data Fig. 1 [file 44319_2026_740_MOESM2_ESM.zip › Figure 1/1A/1A_IF_DXR-Sen.tif]

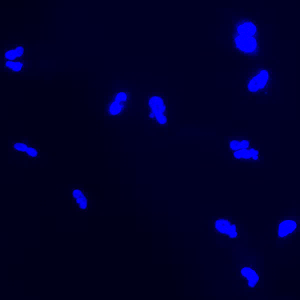

Supplement: Supplementary file 2 — Source data Fig. 1 [file 44319_2026_740_MOESM2_ESM.zip › Figure 1/1A/1A_IF_Rep-Sen.tif]

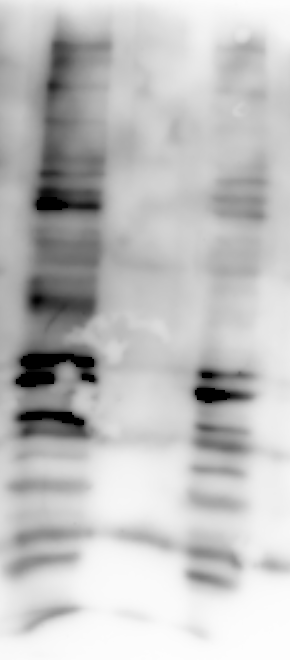

Supplement: Supplementary file 2 — Source data Fig. 1 [file 44319_2026_740_MOESM2_ESM.zip › Figure 1/1A/1A_WB_LMNB1.tif]

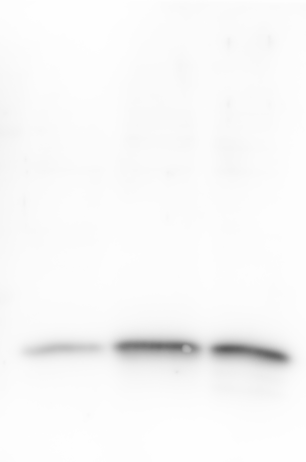

Supplement: Supplementary file 2 — Source data Fig. 1 [file 44319_2026_740_MOESM2_ESM.zip › Figure 1/1A/1A_WB_p16.tif]

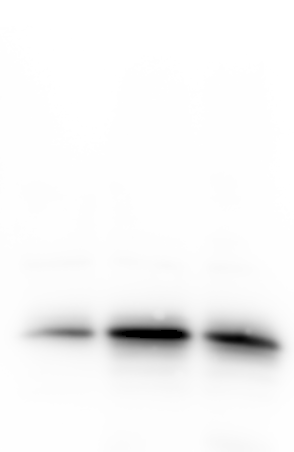

Supplement: Supplementary file 2 — Source data Fig. 1 [file 44319_2026_740_MOESM2_ESM.zip › Figure 1/1A/1A_WB_p21.tif]

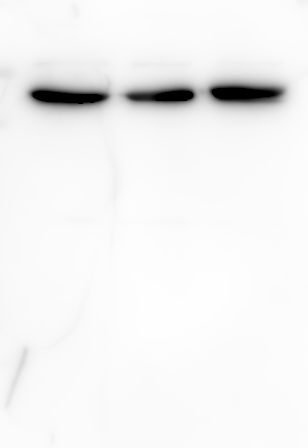

Supplement: Supplementary file 2 — Source data Fig. 1 [file 44319_2026_740_MOESM2_ESM.zip › Figure 1/1A/1A_WB_β-actin.tif]

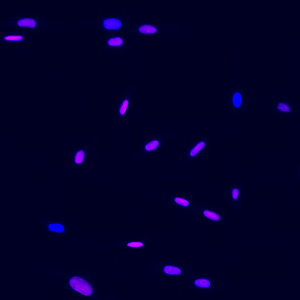

Supplement: Supplementary file 2 — Source data Fig. 1 [file 44319_2026_740_MOESM2_ESM.zip › Figure 1/1C/1C_IF_Control.tif]

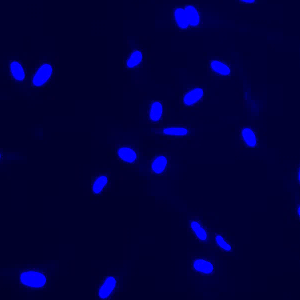

Supplement: Supplementary file 2 — Source data Fig. 1 [file 44319_2026_740_MOESM2_ESM.zip › Figure 1/1C/1C_IF_DXR-Sen.tif]

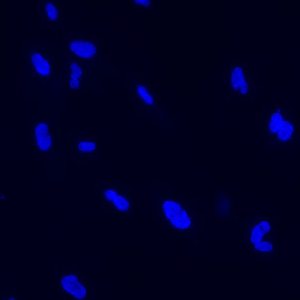

Supplement: Supplementary file 2 — Source data Fig. 1 [file 44319_2026_740_MOESM2_ESM.zip › Figure 1/1C/1C_IF_Rep-Sen.tif]

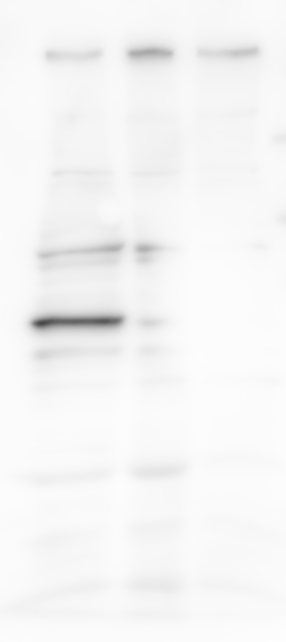

Supplement: Supplementary file 2 — Source data Fig. 1 [file 44319_2026_740_MOESM2_ESM.zip › Figure 1/1C/1C_WB_LMNB1.tif]

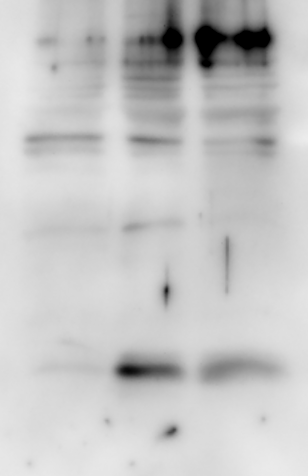

Supplement: Supplementary file 2 — Source data Fig. 1 [file 44319_2026_740_MOESM2_ESM.zip › Figure 1/1C/1C_WB_p16.tif]

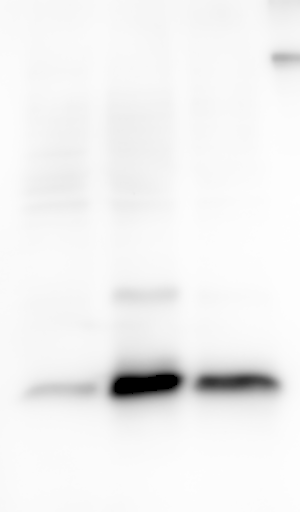

Supplement: Supplementary file 2 — Source data Fig. 1 [file 44319_2026_740_MOESM2_ESM.zip › Figure 1/1C/1C_WB_p21.tif]

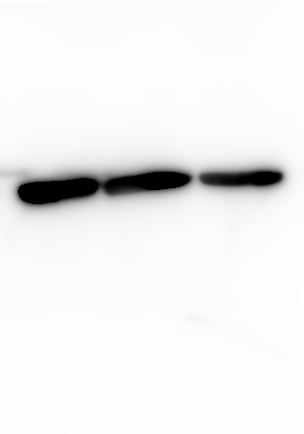

Supplement: Supplementary file 2 — Source data Fig. 1 [file 44319_2026_740_MOESM2_ESM.zip › Figure 1/1C/1C_WB_β-actin.tif]

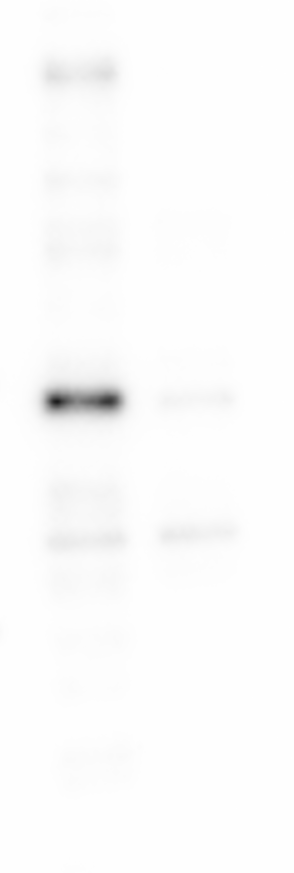

Supplement: Supplementary file 2 — Source data Fig. 1 [file 44319_2026_740_MOESM2_ESM.zip › Figure 1/1E/1E_WB_LMNB1.tif]

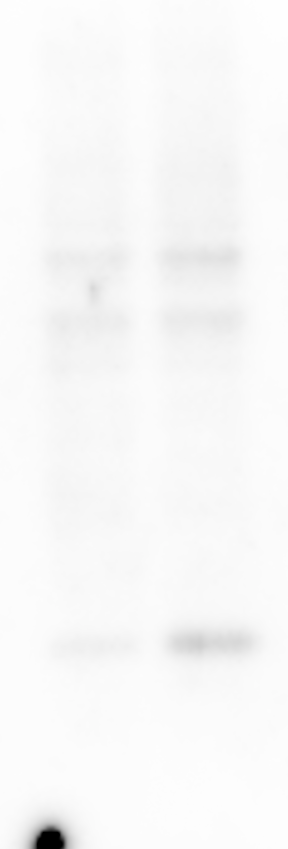

Supplement: Supplementary file 2 — Source data Fig. 1 [file 44319_2026_740_MOESM2_ESM.zip › Figure 1/1E/1E_WB_p16.tif]

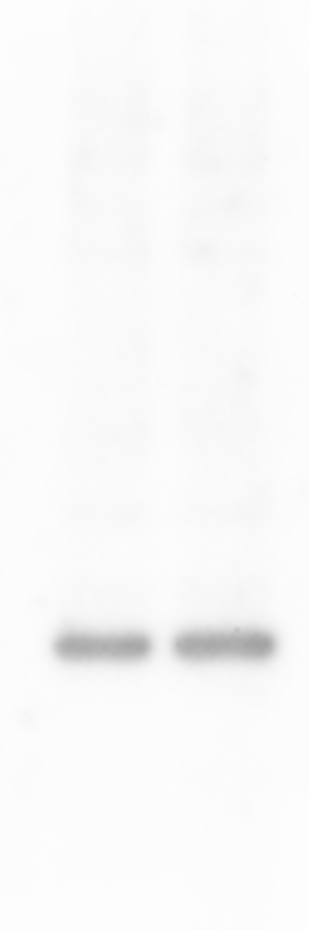

Supplement: Supplementary file 2 — Source data Fig. 1 [file 44319_2026_740_MOESM2_ESM.zip › Figure 1/1E/1E_WB_β-actin.tif]

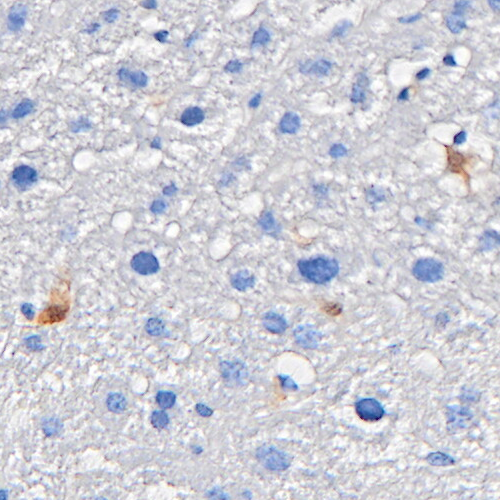

Supplement: Supplementary file 6 — Source data Fig. 5 [file 44319_2026_740_MOESM6_ESM.zip › Figure 5/5D/5D_Liver_aPD-1.tif]

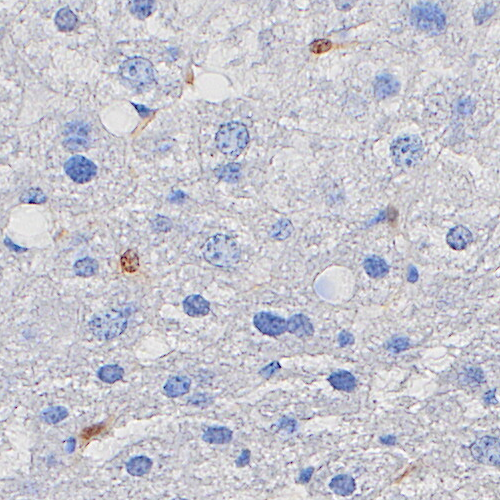

Supplement: Supplementary file 6 — Source data Fig. 5 [file 44319_2026_740_MOESM6_ESM.zip › Figure 5/5D/5D_Liver_Control IgG.tif]

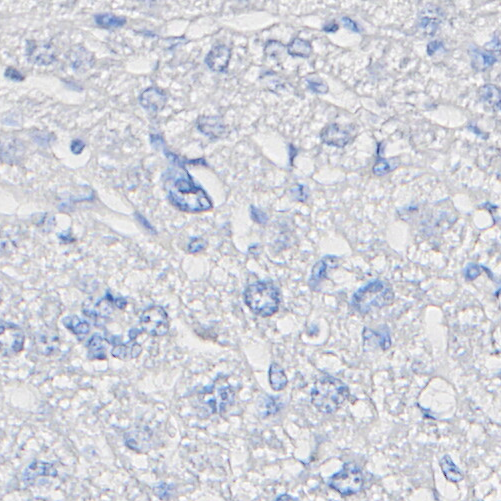

Supplement: Supplementary file 6 — Source data Fig. 5 [file 44319_2026_740_MOESM6_ESM.zip › Figure 5/5D/5D_Liver_p16p21-DKO.tif]

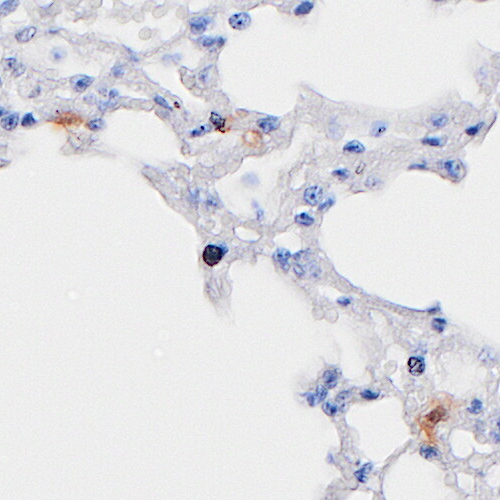

Supplement: Supplementary file 6 — Source data Fig. 5 [file 44319_2026_740_MOESM6_ESM.zip › Figure 5/5D/5D_Lung_aPD-1.tif]

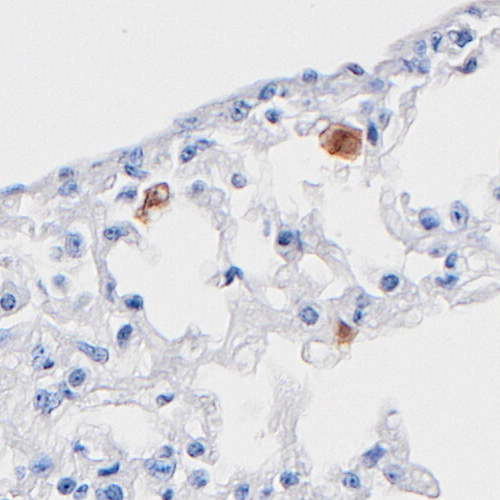

Supplement: Supplementary file 6 — Source data Fig. 5 [file 44319_2026_740_MOESM6_ESM.zip › Figure 5/5D/5D_Lung_Control IgG.tif]

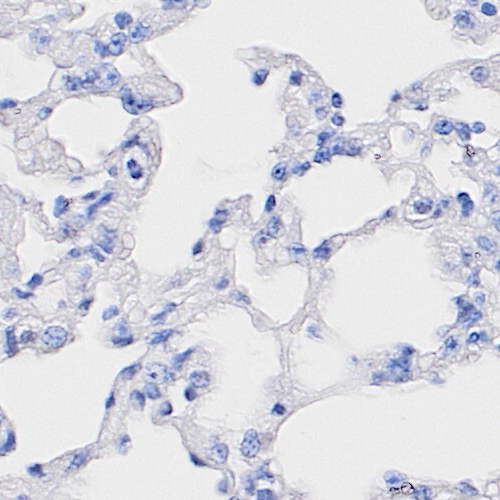

Supplement: Supplementary file 6 — Source data Fig. 5 [file 44319_2026_740_MOESM6_ESM.zip › Figure 5/5D/5D_Lung_p16p21-DKO.tif]

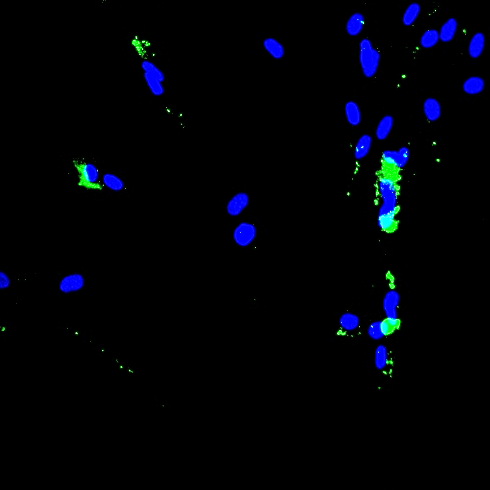

Supplement: Supplementary file 7 — Figure EV1 Source Data [file 44319_2026_740_MOESM7_ESM.zip › Figure EV1/EV1A/EV1A_FC_BPTES_Cayman_10μM.tif]

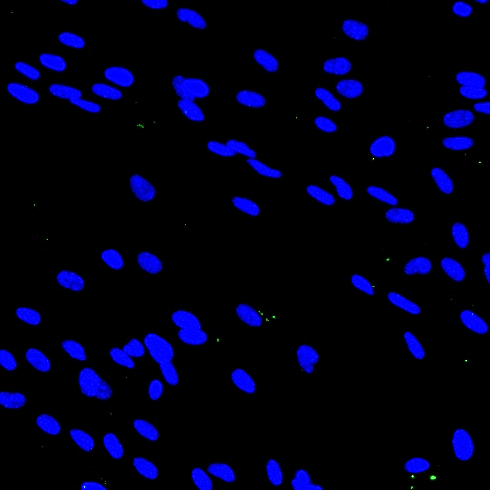

Supplement: Supplementary file 7 — Figure EV1 Source Data [file 44319_2026_740_MOESM7_ESM.zip › Figure EV1/EV1A/EV1A_FC_BPTES_Cayman_1μM.tif]

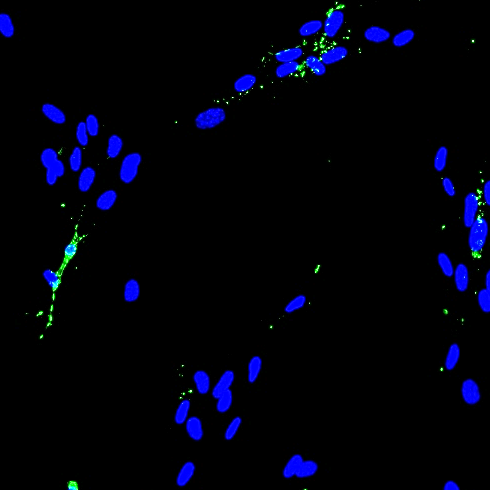

Supplement: Supplementary file 7 — Figure EV1 Source Data [file 44319_2026_740_MOESM7_ESM.zip › Figure EV1/EV1A/EV1A_FC_BPTES_Sigma_10μM.tif]

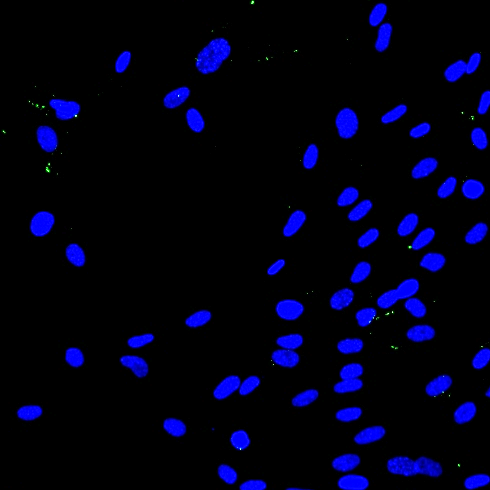

Supplement: Supplementary file 7 — Figure EV1 Source Data [file 44319_2026_740_MOESM7_ESM.zip › Figure EV1/EV1A/EV1A_FC_BPTES_Sigma_1μM.tif]

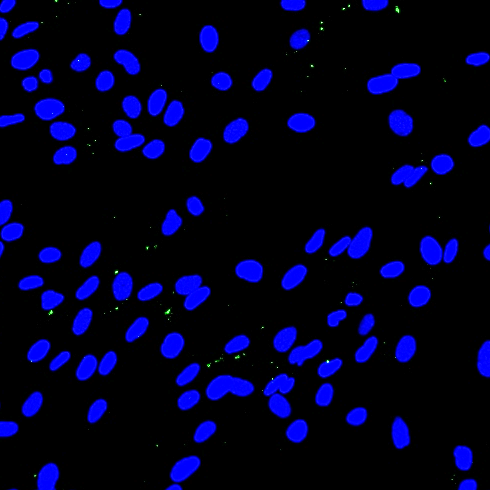

Supplement: Supplementary file 7 — Figure EV1 Source Data [file 44319_2026_740_MOESM7_ESM.zip › Figure EV1/EV1A/EV1A_FC_DMSO.tif]

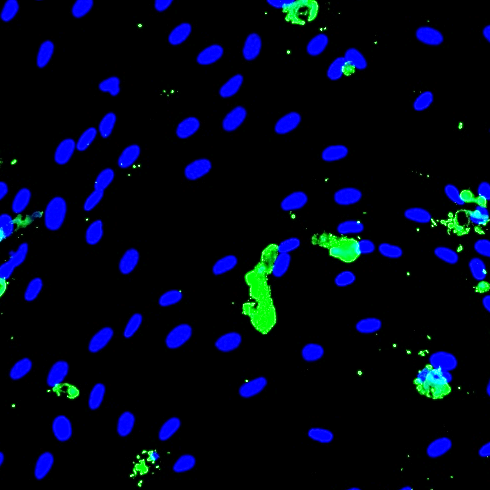

Supplement: Supplementary file 7 — Figure EV1 Source Data [file 44319_2026_740_MOESM7_ESM.zip › Figure EV1/EV1B/EV1B_FC_BPTES_Cayman_10μM.tif]

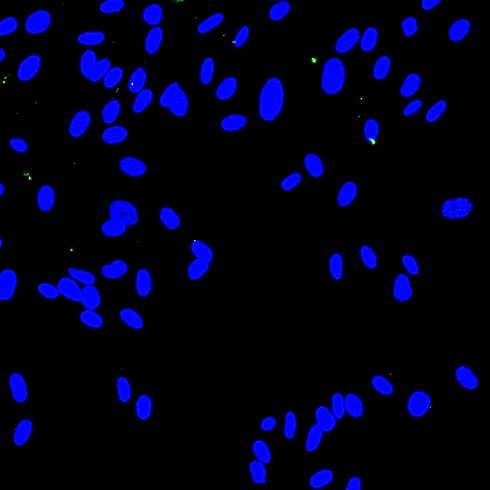

Supplement: Supplementary file 7 — Figure EV1 Source Data [file 44319_2026_740_MOESM7_ESM.zip › Figure EV1/EV1B/EV1B_FC_BPTES_Cayman_1μM.tif]

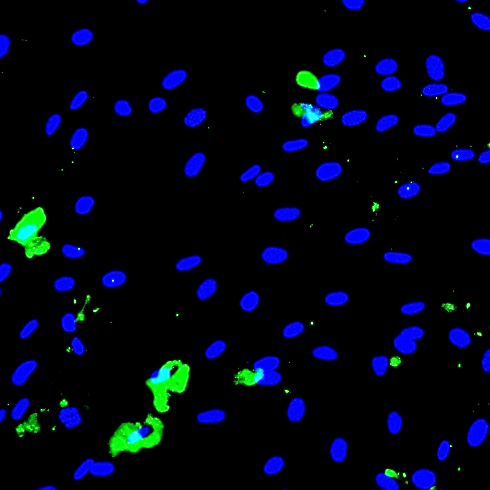

Supplement: Supplementary file 7 — Figure EV1 Source Data [file 44319_2026_740_MOESM7_ESM.zip › Figure EV1/EV1B/EV1B_FC_BPTES_Sigma_10μM.tif]

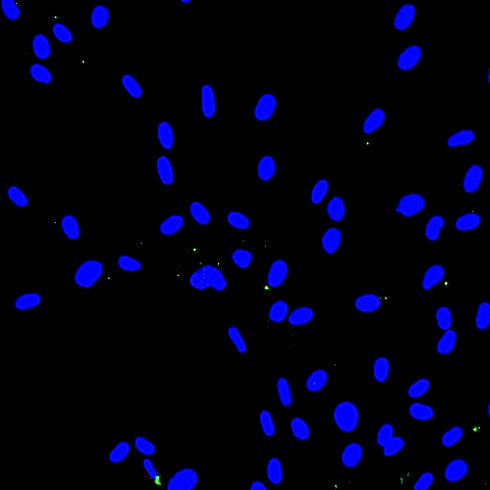

Supplement: Supplementary file 7 — Figure EV1 Source Data [file 44319_2026_740_MOESM7_ESM.zip › Figure EV1/EV1B/EV1B_FC_BPTES_Sigma_1μM.tif]

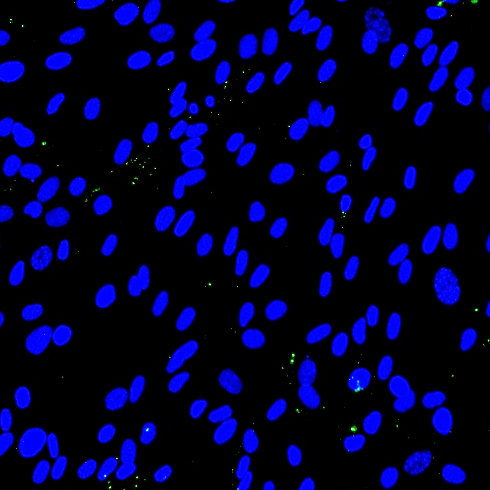

Supplement: Supplementary file 7 — Figure EV1 Source Data [file 44319_2026_740_MOESM7_ESM.zip › Figure EV1/EV1B/EV1B_FC_DMSO.tif]

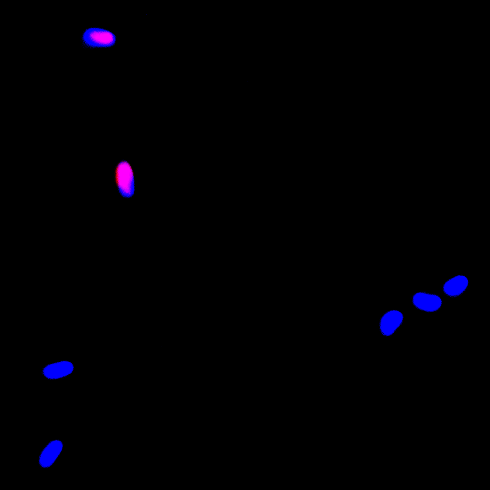

Supplement: Supplementary file 7 — Figure EV1 Source Data [file 44319_2026_740_MOESM7_ESM.zip › Figure EV1/EV1C/EV1C_FC_BPTES_Cayman_10μM.tif]

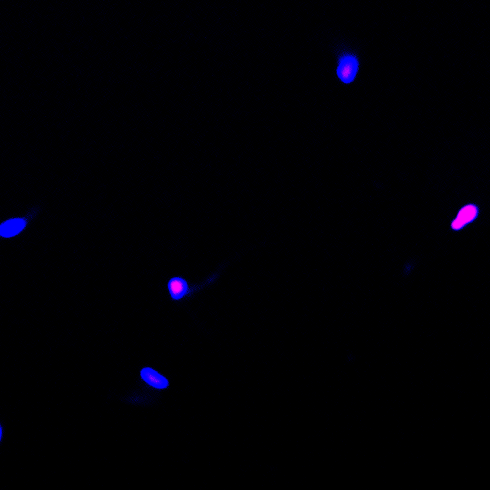

Supplement: Supplementary file 7 — Figure EV1 Source Data [file 44319_2026_740_MOESM7_ESM.zip › Figure EV1/EV1C/EV1C_FC_BPTES_Sigma_10μM.tif]

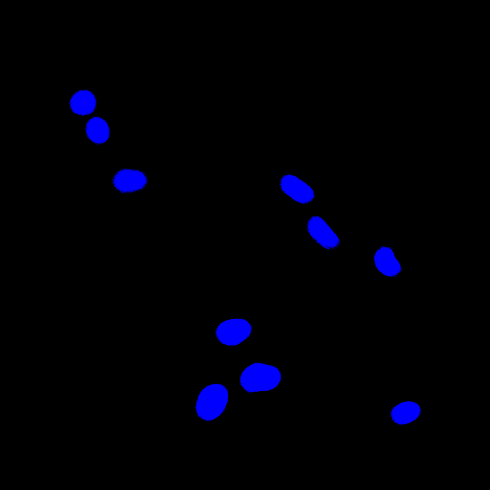

Supplement: Supplementary file 7 — Figure EV1 Source Data [file 44319_2026_740_MOESM7_ESM.zip › Figure EV1/EV1C/EV1C_FC_DMSO.tif]

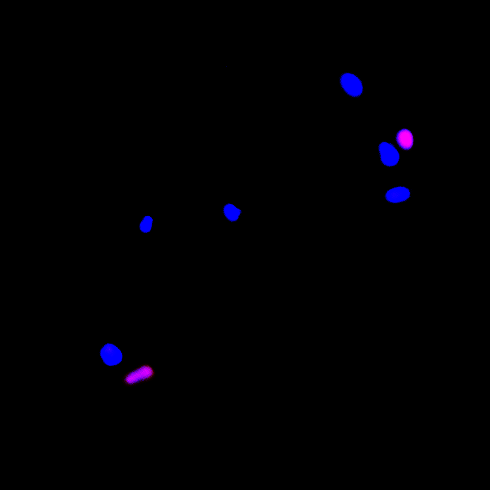

Supplement: Supplementary file 7 — Figure EV1 Source Data [file 44319_2026_740_MOESM7_ESM.zip › Figure EV1/EV1D/EV1D_IF_BPTES_Cayman_10μM.tif]

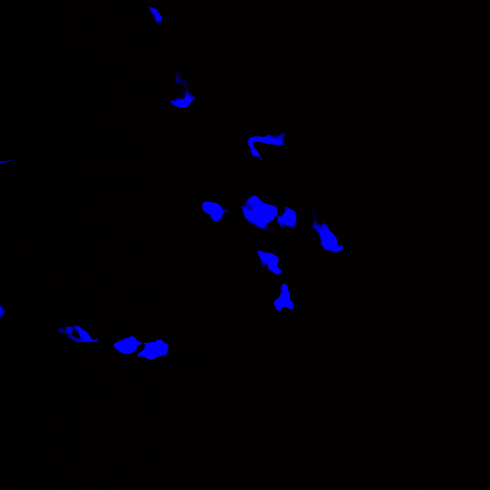

Supplement: Supplementary file 7 — Figure EV1 Source Data [file 44319_2026_740_MOESM7_ESM.zip › Figure EV1/EV1D/EV1D_IF_BPTES_Cayman__1μM.tif]

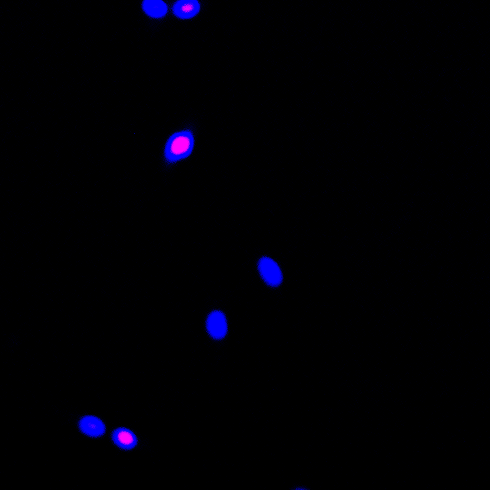

Supplement: Supplementary file 7 — Figure EV1 Source Data [file 44319_2026_740_MOESM7_ESM.zip › Figure EV1/EV1D/EV1D_IF_BPTES_Sigma_10μM.tif]

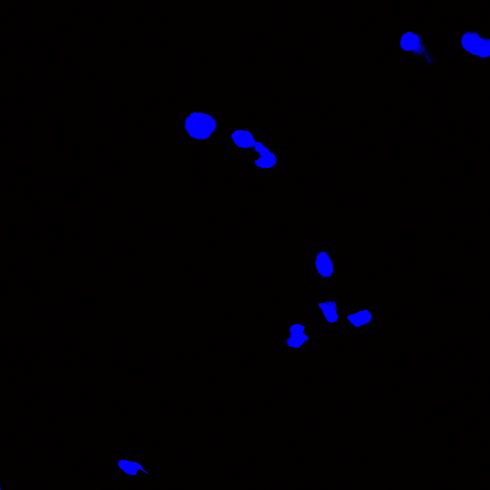

Supplement: Supplementary file 7 — Figure EV1 Source Data [file 44319_2026_740_MOESM7_ESM.zip › Figure EV1/EV1D/EV1D_IF_BPTES_Sigma_1μM.tif]

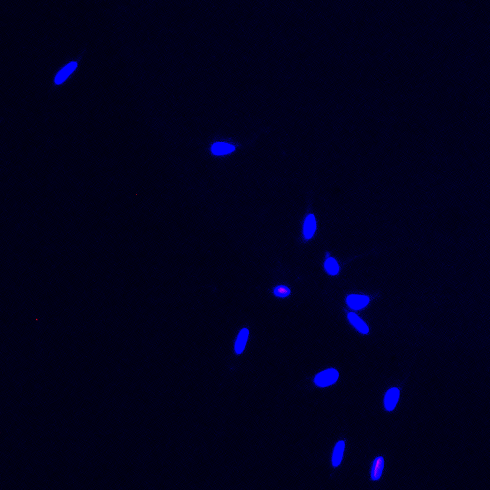

Supplement: Supplementary file 7 — Figure EV1 Source Data [file 44319_2026_740_MOESM7_ESM.zip › Figure EV1/EV1D/EV1D_IF_DMSO.tif]
