# Supplementary material for: Reevaluating the senolytic activity of a GLS1 inhibitor and an anti-PD-1 antibody: toward greater reproducibility and methodological rigor
Source: EMBO Rep. 2026 Apr 3;27(9):2201–17. doi: 10.1038/s44319-026-00740-5 (PMC13172396; doi:10.1038/s44319-026-00740-5)
Supplement: Supplementary file 9 — Expanded View Figures [file 44319_2026_740_MOESM9_ESM.pdf]

Expanded View Figures

Figure EV1. High dose of BPTES elicit apoptosis in control HDFs.

(**A–D**) Non-senescent early-passage HDFs, IMR-90 (**A**, **C**) and TIG-3 (**B**, **D**), were treated with BPTES from Sigma-Aldrich or Cayman at the indicated concentrations for 2 days. Apoptotic cells positive for fluorochrome-labeled annexin V or TUNEL were visualized by fluorescence microscopy, and the proportions of annexin V- or TUNEL-positive cells were quantified. Data are presented as mean ± s.d. (**A–D**, $n = 4$). All of the experiments were repeated at least twice, independently, with similar results. Statistical significance was determined by one-way ANOVA followed by Sidak's test. DMSO vs. BPTES (Sigma) 10 mM: $^{**}p = 0.0018$ (IMR-90), $^{***}p = 0.0002$ (TIG-3); DMSO vs. BPTES (Cayman) 10 μM: $^{****}p < 0.0001$ (IMR-90, TIG-3); BPTES (Sigma) 1 vs. 10 μM: $^{**}p = 0.0016$ (IMR-90), $^{***}p = 0.0002$ (TIG-3); BPTES (Cayman) 1 vs. 10 μM: $^{****}p < 0.0001$ (IMR-90, TIG-3) (**A**, **B**). DMSO vs. BPTES (Sigma) 10 μM: $^{***}p = 0.0001$ (IMR-90), $^{****}p < 0.0001$ (TIG-3); DMSO vs. BPTES (Cayman) 10 μM: $^{***}p = 0.0002$ (IMR-90), $^{***}p = 0.0006$ (TIG-3); BPTES (Sigma) 1 vs. 10 μM: $^{***}p = 0.0003$ (IMR-90), $^{***}p = 0.0001$ (TIG-3); BPTES (Cayman) 1 vs. 10 μM: $^{***}p = 0.0003$ (IMR-90), $^{***}p = 0.0006$ (TIG-3) (**C**, **D**). Scale bars, 10 μm. Source data are available online for this figure.

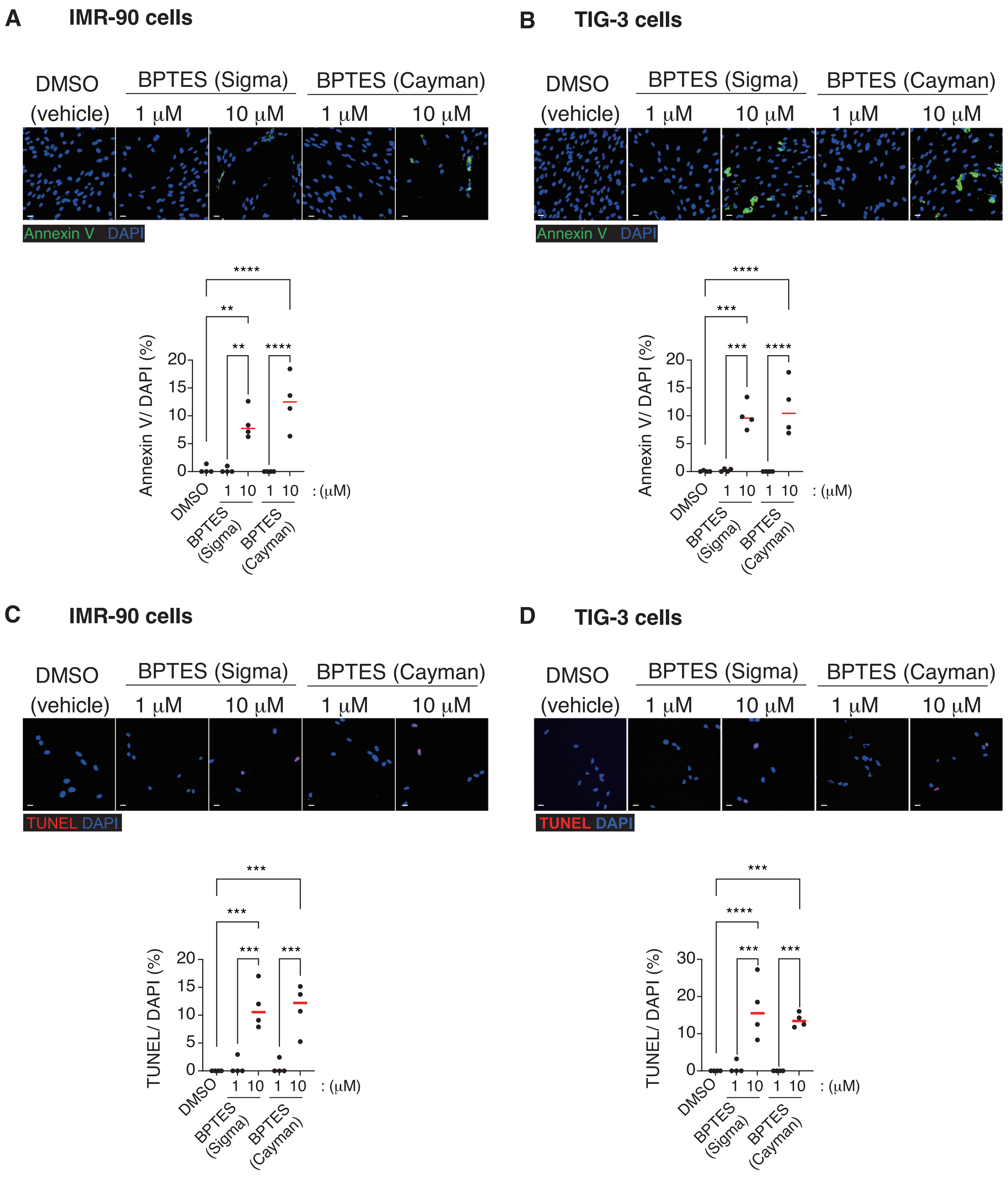
A
IMR-90 cells
DMSO (vehicle)
BPTES (Sigma)
BPTES (Cayman)
1 µM
10 µM
1 µM
10 µM
Annexin V DAPI
Annexin V/ DAPI (%)
0
5
10
15
20
****
**
**
****
DMSO
1 10
1 10
: (µM)
BPTES (Sigma)
BPTES (Cayman)
B
TIG-3 cells
DMSO (vehicle)
BPTES (Sigma)
BPTES (Cayman)
1 µM
10 µM
1 µM
10 µM
Annexin V DAPI
Annexin V/ DAPI (%)
0
5
10
15
20
****
***
***
****
DMSO
1 10
1 10
: (µM)
BPTES (Sigma)
BPTES (Cayman)
C
IMR-90 cells
DMSO (vehicle)
BPTES (Sigma)
BPTES (Cayman)
1 µM
10 µM
1 µM
10 µM
TUNEL DAPI
TUNEL/ DAPI (%)
0
5
10
15
20
***
***
***
***
DMSO
1 10
1 10
: (µM)
BPTES (Sigma)
BPTES (Cayman)
D
TIG-3 cells
DMSO (vehicle)
BPTES (Sigma)
BPTES (Cayman)
1 µM
10 µM
1 µM
10 µM
TUNEL DAPI
TUNEL/ DAPI (%)
0
10
20
30
***
****
***
***
DMSO
1 10
1 10
: (µM)
BPTES (Sigma)
BPTES (Cayman)

© The Author(s)

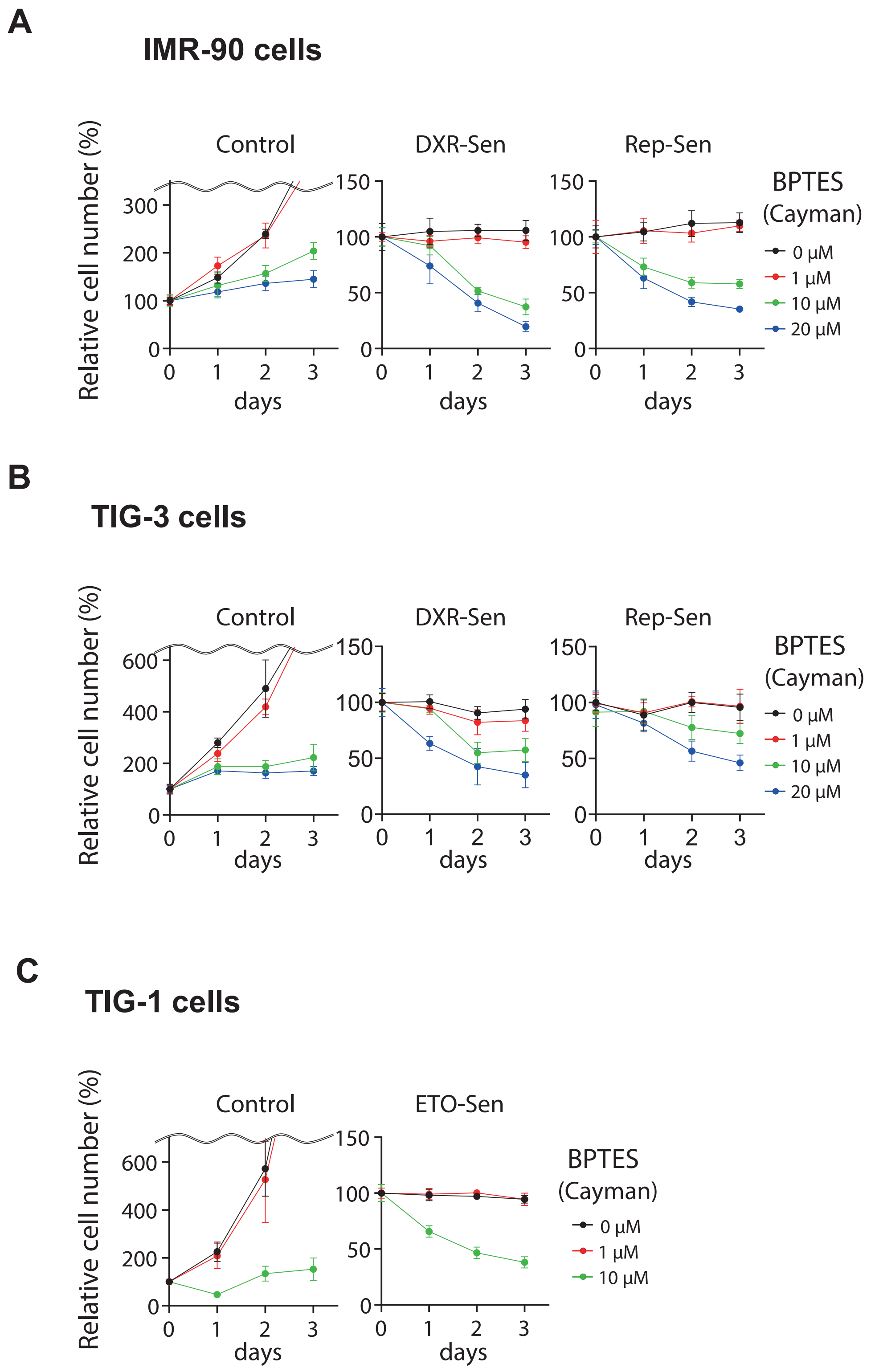

Figure EV2. Effect of BPTES from different suppliers on proliferation and survival of senescent HDFs.

(**A–C**) Control and senescent HDFs were treated with BPTES from Cayman at the indicated concentrations for 3 days. Cell numbers were monitored throughout the experimental period, and relative cell numbers were quantified. Data are presented as mean ± s.d. (**A**, **B**: $n = 4$; **C**: $n = 3$). All of the experiments were repeated at least twice, independently, with similar results. Source data are available online for this figure.
